# Prevalence of hepatitis B in Latin America and the Caribbean: a systematic review and meta-analysis

**DOI:** 10.1007/s00705-026-06562-z

**Published:** 2026-02-21

**Authors:** Mariana Cavalheiro Magri, Caroline Manchiero, Bianca Peixoto Dantas, Wanderley Marques Bernardo, Edson Abdala, Fátima Mitiko Tengan

**Affiliations:** 1 Laboratorio de Investigacao Medica em Hepatologia por Virus (LIM-47), Hospital das Clinicas HCFMUSP, Faculdade de Medicina, Universidade de Sao Paulo, Av. Dr. Enéas de Carvalho Aguiar, 470 - Cerqueira Cesar, Zip code: 05403-000 Sao Paulo, SP Brazil; 2 Instituto de Medicina Tropical de Sao Paulo, Faculdade de Medicina, Universidade de Sao Paulo, Sao Paulo, Brazil; 3 Faculdade de Medicina, Universidade de Sao Paulo, Sao Paulo, Brazil; 4 Departamento de Infectologia e Medicina Tropical, Faculdade de Medicina, Universidade de Sao Paulo, Sao Paulo, Brazil

## Abstract

Hepatitis B is a health threat with regional variations in prevalence. Our aim was to conduct a systematic review and meta-analysis of available data on the prevalence of hepatitis B virus (HBV) in Latin America and the Caribbean (LAC). A literature search was conducted from 2000 to 2024 using the databases Medline, Embase, LILACS and Web of Science, following PRISMA guideline. Search terms included: “hepatitis B virus”, “HBsAg”, “HBV”, “prevalence”, and “Latin America and the Caribbean”. Random-effects meta-analysis was performed to estimate the prevalence of HBV (HBsAg), and the *I*^2^ statistic was used to assess heterogeneity between studies. Subgroup and meta-regression analyses were performed to investigate possible sources of heterogeneity. In total, 190 studies were included in the analysis and the estimated global prevalence of HBV was 1.0% (95% CI: 1.0%-1.0%). In population subgroup analyses, the prevalence in general population was 1.0%, indigenous people was 6.0%, HIV-infected individuals was 5.0%, and inmates was 1.0%. The prevalence found was 2.0% in the period of 2000–2008, 1.0% in 2009–2016, and 1.0% in 2017–2024. The prevalence trends over time in the general population, indigenous people, HIV-infected individuals and inmate subgroups were 1.0%, 11.0%, 6.0% and 12.0% in the period of 2000–2008, 1.0%, 4.0%, 3.0% and 0.0% in 2009–2016, and 1.0%, 5.0%, 8.0% and 0.1% in 2017–2024, respectively. In LAC, HBV prevalence decreases over time, but risk subgroups stand out with elevated prevalence, such as indigenous people and HIV-infected individuals. Strategies to expand testing, treatment and vaccination with a focus on these subgroups may be relevant to reduce the prevalence of HBV.

**Supplementary Information:**

The online version contains supplementary material available at 10.1007/s00705-026-06562-z.

## Introduction

Hepatitis B remains a significant health threat, with approximately 254 million people living with the disease worldwide, but regional variations may occur, with high prevalence in Africa and the Western Pacific regions [1]. Hepatitis B is caused by the hepatitis B virus (HBV), a member of the family *Hepadnaviridae a*nd the genus *Orthohepadnavirus*. Although it is usually an acute self-limited disease in adults, it has long been recognized as a cause of chronic disease leading to liver damage, cirrhosis, and hepatocellular carcinoma [2]. Severe HBV infection during pregnancy poses high risks for maternal and fetal outcomes. Gestational immunological and hormonal changes may exacerbate liver injury and increase the risk of fulminant hepatitis [3, 4]. In these scenarios, prognosis can be assessed using the MELD scoring system [4], and management requires early diagnosis, supportive therapy, and timely obstetric intervention to mitigate maternal mortality [5].

Not all individuals diagnosed with chronic hepatitis B have access to treatment. When available, treatments include reverse transcriptase inhibitors, which are nucleot(s)ide analogues that potently suppress HBV replication (e.g., tenofovir and entecavir), with excellent clinical results and with hepatitis B surface antigen (HBsAg) loss in serum. However, it requires lifelong treatment, and drug resistance may develop. Additionally, current therapy may also include immunomodulators (e.g., interferon) [6]. There is insufficient data to establish which patients would actually benefit from treatment stopping [7]. Complete cure is challenging, mainly because of the covalently closed circular DNA (cccDNA) of HBV persistence [8]. Efforts are required for new curative therapies, which may include capsid-assembly inhibitors and also immune-stimulating approaches [9].

However, evidence suggests that only 13% of people living with chronic hepatitis B infection worldwide had been diagnosed [1]. HBV screening can be performed through serological testing, which is capable of detecting and quantifying viral-specific antibodies and/or antigens. These markers allow the identification of patients with infection and characterize the phases of hepatitis B [10, 11]. For instance, the presence of HBsAg is a valuable marker of an ongoing infection, and its presence for at least six months defines chronic infection [12]. HBV-DNA can be mostly detected by molecular tests such as real-time polymerase chain reaction (PCR) and nucleic acid testing (NAT). The DNA quantification is fundamental for assessing viral load, which may imply the risk of viral transmission, disease progression, and evaluation of therapy effectiveness [11, 13]. Additionally, liver function tests, such as alanine aminotransferase (ALT) levels in blood, can indicate inflammation and are an additional test in the diagnosis and monitoring of hepatitis B [13].

Diagnosing hepatitis of unknown origin remains a major challenge, particularly in children, as occult viral etiologies may remain undetected even in severe cases [14]. The risk of intrauterine HBV transmission is influenced by high maternal HBV DNA levels; however, accurate diagnosis is difficulted by test limitations, placental barriers, and viral latency [15]. Furthermore, infection phases such as the window period, occult HBV infection, low-level viremia and surface antigen escape mutants can yield false-negative HBsAg results, further complicating diagnosis [11]. HBV is mainly transmitted through sexual, perinatal, and parenteral routes, with chronic infection risk decreasing with age. Populations at highest risk include individuals from endemic regions, people who inject drugs, men who have sex with men, and close contacts of HBsAg-positive individuals [16].

Prognostic assessment in chronic hepatitis B depends on both viral and host factors. The HBsAg is a key marker of active and persistent infection, and its quantitative measurement plays a critical role in evaluating treatment prognosis and determining HBV chronicity. Prognostic predictors include HBV DNA levels, which provide insights into the risk of hepatocellular carcinoma, while the anti-HBe seroconversion are associated with more favorable outcomes. Combining these and other biomarkers with noninvasive fibrosis scores, such as FIB-4 or APRI, improves risk stratification and prognostic accuracy. Additional tools, including viral genotyping and imaging assessments, are also relevant [13].

Vaccination is a safe and effective intervention and remains an essential preventive measure against hepatitis B. It should be administered within the first 24 h after birth followed by an additional 2–3 doses [1, 17]. In the Americas, the hepatitis B vaccine was progressively incorporated into childhood immunization programs between 1991 and 2012, with phased implementation across Latin America and the Caribbean (LAC) countries: Cuba and Colombia (1994), French Guiana and the Dominican Republic (1994), Brazil (1998), Mexico and Uruguay (1999), Argentina, Bolivia, Honduras, and Venezuela (2000), Jamaica and Peru (2003), Chile and Suriname (2005). Several countries, including Argentina, Brazil, Cuba, Peru, Uruguay, and the United States, have also extended vaccination to older age groups as a complementary control strategy [18].

There are many challenges to be overcome to reduce the number of people infected with HBV, such as diagnosing it and be aware of its prevalence. Many systematic reviews with meta-analyses on the prevalence of hepatitis B are available. However, there are few studies on the topic in LAC that emphasize risk groups for which interventions can be targeted. Our aim was to conduct a systematic review and meta-analysis of available data on the prevalence of HBV in LAC.

## Methods

This systematic review followed the Preferred Reporting Items for Systematic Review and Meta-Analysis (PRISMA) guidelines. The review protocol was registered in the International Prospective Register of Systematic Reviews (PROSPERO, https://www.crd.york.ac.uk/PROSPERO) under the registration number CRD42024559720.

### Data sources

The searches were conducted in Medline through the Pubmed platform, Embase, Latin American and Caribbean Literature (LILACS) and Web of Science databases. The terms “hepatitis B virus”, “HBsAg”, “HBV”, “prevalence”, “Latin America and the Caribbean” or a combination of them were used. In the Medline, Embase and Web of Science databases, we searched for publications from the year 2000 up to June 2024, without language restrictions. In the LILACS database, there were no time or language restrictions. Details are described in the Supplementary Material S1. We also searched for relevant studies in the bibliographic references of the selected studies and review articles on the topic. Disagreements in identifying relevant studies were discussed until consensus was reached.

### Study selection criteria

We looked for full articles that provided an estimate prevalence of HBV in LAC, with a sample size greater than or equal to 50. We did not select case reports, review articles, commentaries, studies whose participants do not reside in Latin America or in the Caribbean, or studies that contain the same case series presented in other publications. Regarding the latest studies, we included the article with the most complete data.

We excluded studies that consider self-reported HBV infection, data obtained from mandatory HBV reporting (e.g., local Ministry of Health databases), and studies whose participant selection method was unclear.

The following definition was used:

- HBV infection: presence of HBsAg through serological or rapid diagnostic tests.

### Data extraction

The information listed below were extracted from the studies: name(s) of the author(s), year of publication, country, cohort, sample size, mean age, percentage of male participants and number of HBsAg-positive individuals. Data collection was conducted independently by two researchers, whose differences were resolved through discussion and consensus.

### Assessment of the quality of studies

To assess the methodological quality of selected studies, we used the Joanna Briggs Institute (JBI) Critical Appraisal Checklist for Studies Reporting Prevalence Data. The instrument offers nine items that can be scored as positive or negative. Studies with scores of 0 to 3, 4 to 6, and 7 to 9 represent high, moderate and low risk of bias, respectively. Disagreements were discussed between the two researchers who, independently, carried out the risk assessment.

### Statistical analysis

All analyses were performed using the random effects model, which considers variation between studies. Heterogeneity was assessed by the *I*^*2*^ statistic, which shows mild, moderate and high heterogeneity between studies: 25–50%, 51–75% and > 75%, respectively. Subgroup and meta-regression analyzes were performed to investigate possible sources of heterogeneity.

We analyzed in particular the subgroups: general population, including sentinel populations such as blood donors and pregnant women, human immunodeficiency virus (HIV), indigenous people and inmates.

We used meta-regression to investigate the effect of sample size (continuous variable), year of study publication (continuous variable), and study quality (continuous variable) on the prevalence of HBV. Publication bias was examined by using a test to detect asymmetry in the funnel plot proposed by Begg and Mazumdar [19] and the test by Egger et al. [20]. Additionally, we performed three sensitivity analyses, excluding: (1) studies where the study quality assessment score was ≤ 5, (2) studies where the sample size was ≤ 200 and (3) studies where the sample size was ≤ 500.

Furthermore, we estimated, through meta-analysis, the prevalence of HBV in the total study population and in population subgroups in three time periods: 2000–2008, 2009–2016, and 2017–2024.

All analyzes were performed using Stata software version 16 (Stat Corp LP, Texas USA).

## Results

**Fig. 1:**
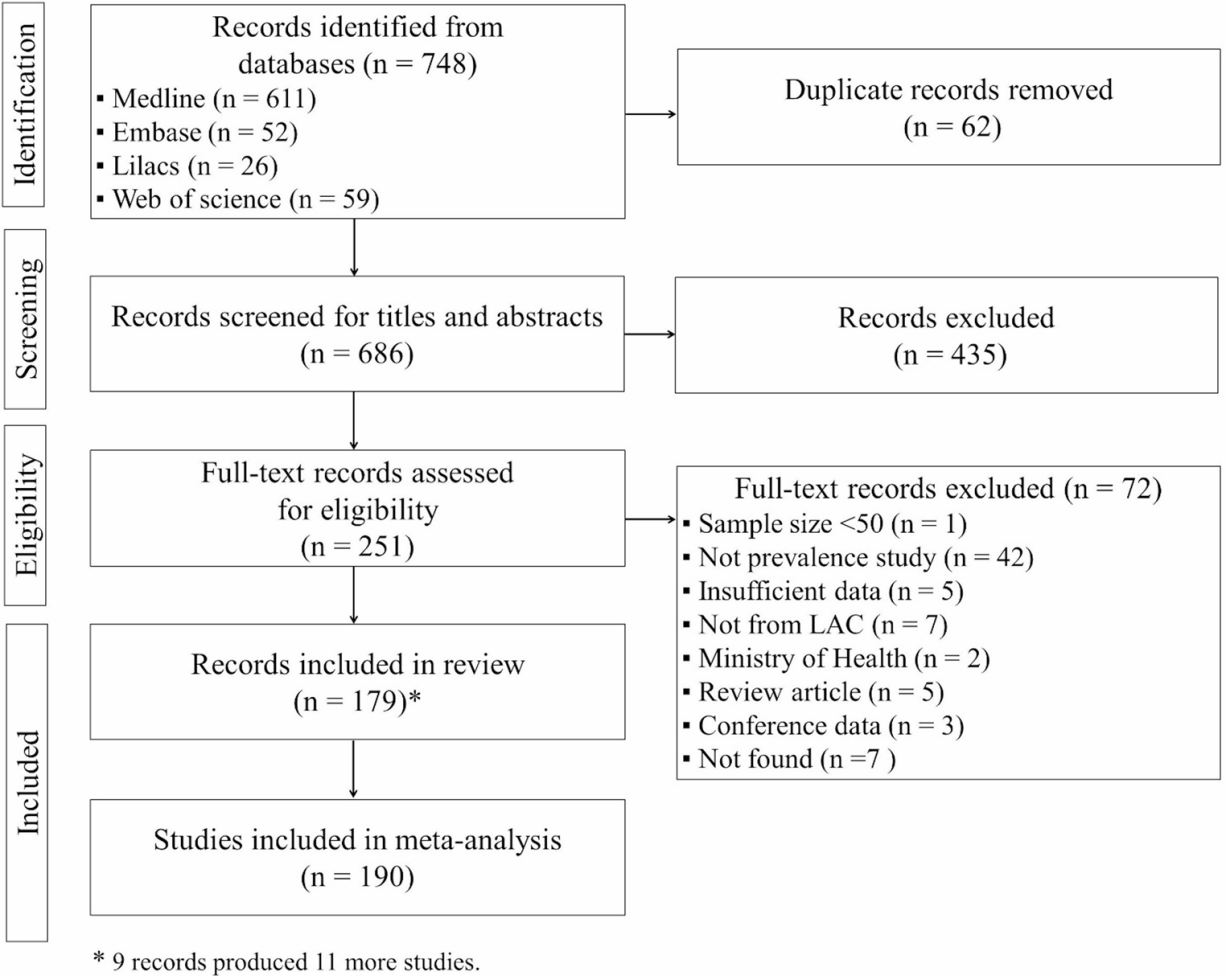
Flow diagram of the screening and selection processes for the meta-analysis of the prevalence of HBV in Latin America and the Caribbean

After searching the Medline, Embase, Web of Science and LILACS databases, with the aim of finding relevant scientific literature on the prevalence of HBV in LAC, we identified 686 references, after excluding duplicates (Fig. 1). After reading titles and abstracts of references and excluding 435 citations, we selected 251 relevant articles. After reading the full text of these 251 articles, we selected 179 references. As nine articles gave rise to two or more studies, contributing to eleven more studies, 190 studies remained for analysis (Supplementary Material S2). As an example, Jose-Abrego et al. [21] reported the study of the prevalence of HBV in chronic liver disease, people living with HIV/AIDS and indigenous people in the same publication, totaling three studies in their article.

A hundred and eighteen studies (62.1%) were conducted in Brazil, 18 (9.5%) in Mexico, 13 (6.8%) in Argentina, 12 (6.3%) in Peru, seven (3.7%) in Venezuela, four (2.1%) in Chile, four (2.17%) in Colombia, three (1.6%) in Jamaica, two (1.1%) in Bolivia, two (1.1%) in French Guiana, one of each (0.5%) in Cuba, Dominican Republic, Guadeloupe, Suriname, Honduras and Uruguay. The sample size ranged from 50 to 1,142,228 (mean 13.884; median 486). Twenty-four studies (12.6%) presented a low risk of bias, 163 (85.8%) a moderate risk and three (1.6%) a high risk of bias.

**Fig. 2:**
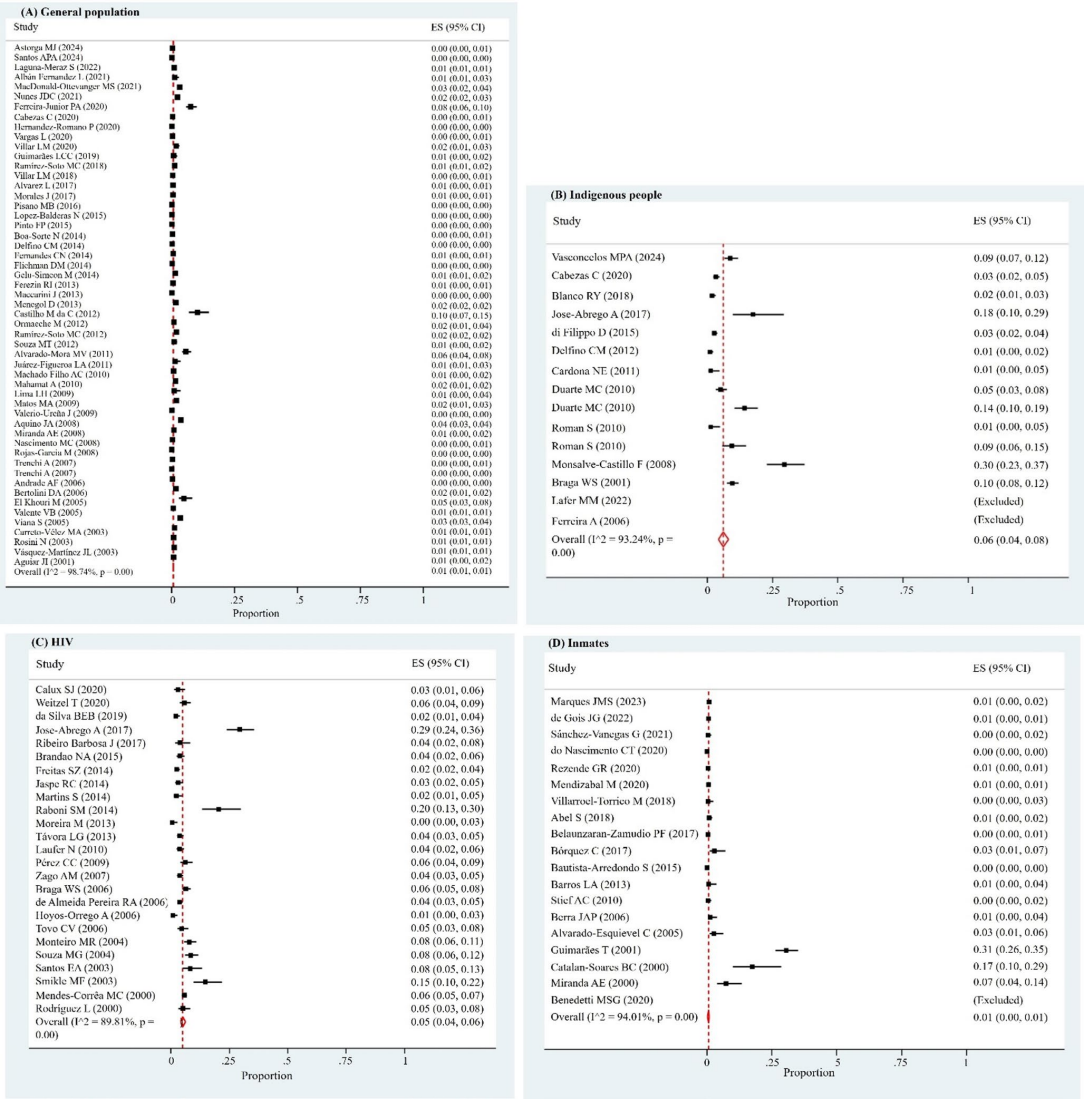
Forest plot showing the estimated global prevalence of HBV in Latin America and the Caribbean according to population subgroups: (**A**) general population, (**B**) indigenous people, (**C**) human immunodeficiency virus (HIV)-infected individuals, and (**D**) inmates

The prevalence of HBV (defined as the presence of HBsAg) ranged from 0.0% to 30.0%, and the overall prevalence, obtained through meta-analysis of selected studies, was 1.0% (95% CI: 1.0%-1.0%), with considerable heterogeneity (*I*^*2*^ = 97.29%, *p* = 0.00) (Supplementary Material S3). In population subgroup analyses, we obtained prevalences that varied from 1.0% (general population and inmates) to 6.0% (indigenous people) (Table 1; Fig. 2).

**Table 1:** Subgroup analysis of the prevalence of HBV in Latin America and the Caribbean

Subgroups	Number of studies	Sample size	Estimated prevalence of HBsAg (%)	95% CI
Period of publication				
Entire period	190	2,638,016	1.0	1.0–1.0
2000 to 2008	60	509,698	2.0	2.0–2.0
2009 to 2016	74	825,260	1.0	1.0–1.0
2017 to 2024	56	1,303,058	1.0	1.0–1.0
Study population				
General population	53	2,496,980	1.0	1.0–1.0
2000 to 2008	15	480,091	1.0	1.0–1.0
2009 to 2016	22	776,123	1.0	0.0–1.0
2017 to 2024	16	1,240,766	1.0	1.0–1.0
Indigenous people	15	5,654	6.0	4.0–8.0
2000 to 2008	3	1,051	11.0	9.0–13.0
2009 to 2016	7	2,524	4.0	2.0–6.0
2017 to 2024	5	2,079	5.0	2.0–9.0
HIV	25	11,578	5.0	4.0–6.0
2000 to 2008	11	6,206	6.0	4.0–7.0
2009 to 2016	9	3,924	3.0	2.0–5.0
2017 to 2024	5	1,448	8.0	3.0–13.0
Inmates	19	67,493	1.0	0.0–1.0
2000 to 2008	5	1,015	12.0	2.0–21.0
2009 to 2016	3	17,640	0.0	0.0–0.0
2017 to 2024	11	48,838	0.1	0.0-0.1

HBsAg: hepatitis B surface antigen, HIV: human immunodeficiency virus, CI: confidence interval

Sample size and year of study publication were inversely associated with the intensity of the effect in the meta-regression analysis (*p* = 0.002 and *p* = 0.001, respectively) (Supplementary Material S4). The funnel plot appeared to be asymmetrical and the Egger and Begg tests were statistically significant in detecting publication bias (Supplementary Material S5).

**Fig. 3:**
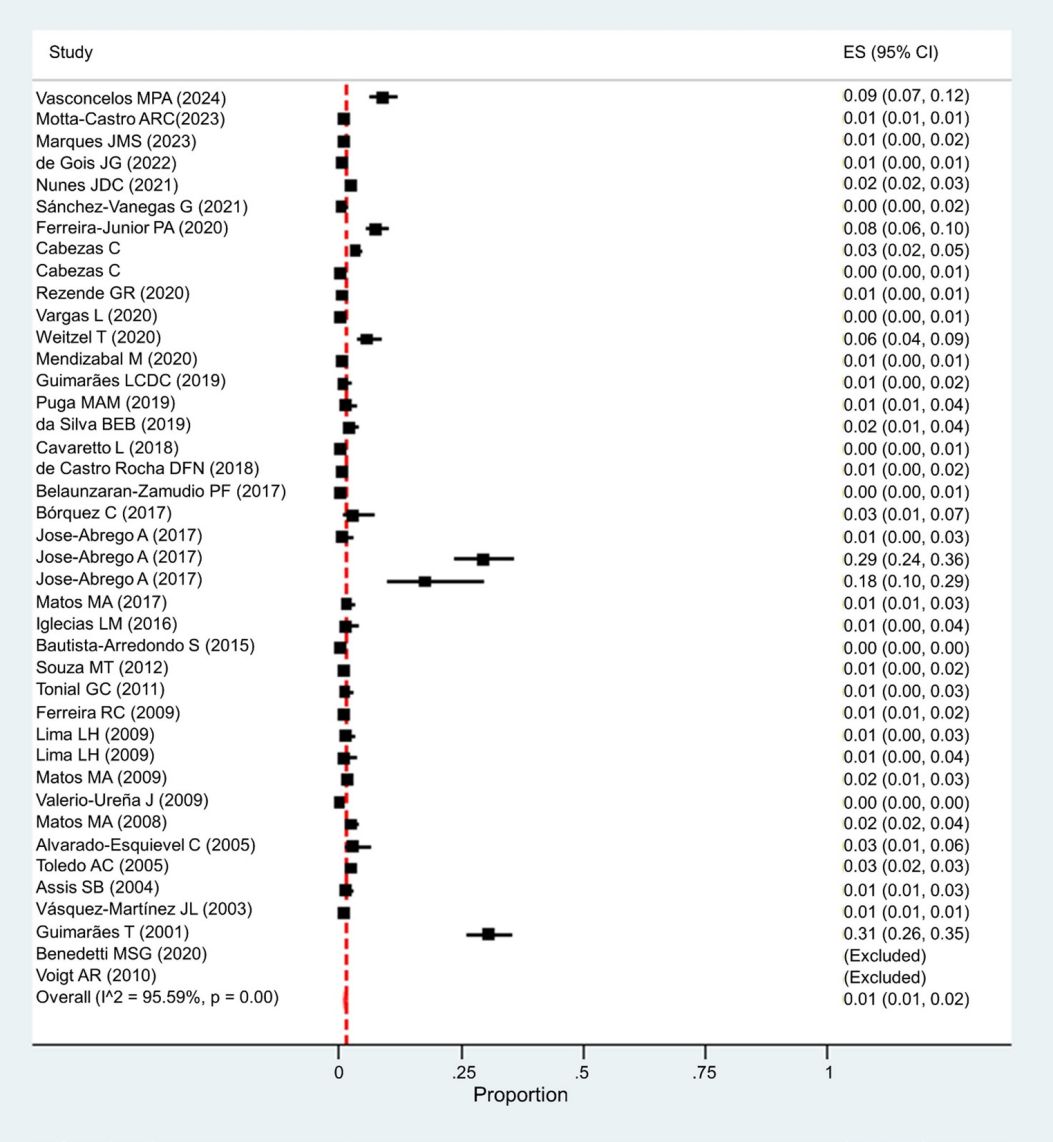
Forest plot showing the estimated prevalence of HBV in Latin America and the Caribbean according to sensitivity analyses including studies with methodological quality greater than 5

In the meta-analysis of studies with a sample size greater than 200 and greater than 500, we found a prevalence of HBV of 1.0% (95% CI: 1.0–1.0) in both (Supplementary Material S6). In addition, when we analyzed studies with methodological quality greater than 5, a prevalence of 1.0% (95% CI: 1.0–2.0) was found (Fig. 3).

A meta-analysis was also conducted on the studies selected by period of publication: in the first period (2000 to 2008) the prevalence of HBV found was 2.0% (95% CI: 2.0–2.0), in the second (2009 to 2016) it was 1.0% (95% CI: 1.0–1.0) and in the third (2017 to 2024) it was 1.0% (95% CI: 1.0–1.0) (Table 1 and Supplementary Material S7). Additional meta-analysis was conducted on the subgroups by period of publication. The prevalence trends over time in the general population, indigenous people, HIV-infected individuals and inmate subgroups were 1.0% (95% CI: 1.0–1.0), 11.0% (95% CI: 9.0–13.0), 6.0% (95% CI: 4.0–7.0), and 12.0% (95% CI: 2.0–21.0) in the first period (2000 to 2008), was 1.0% (95% CI: 0.0–1.0), 4.0% (95% CI: 2.0–6.0), 3.0% (95% CI: 2.0–5.0), and 0.0% (95% CI: 0.0–0.0) in the second (2009 to 2016), and 1.0% (95% CI: 1.0–1.0), 5.0% (95% CI: 2.0–9.0), 8.0% (95% CI: 3.0–13.0), and 0.1% (95% CI: 0.0-0.1) in the third (2017 to 2024), respectively (Table 1, Supplementary Material S8, S9, S10 and S11).

## Discussion

We systematically evaluated the prevalence of HBV (presence of HBsAg) in LAC in the last 24 years. The 190 studies included in the meta-analysis revealed an estimated global prevalence of 1.0%. The majority of these studies were from Brazil, Mexico and Argentina.

According to the Global Burden of Disease Study 2019 report [22], the global prevalence of HBV across all ages was 4.1%. In South American countries, the highest overall prevalences of HBV were detected in Brazil (3.4%) and Paraguay (3.0%), while the lowest prevalences were detected in Argentina and Uruguay (0.3% for both). In the present review, the studies reporting the highest prevalences of HBV were from Venezuela (29.5% in indigenous people) [23], Mexico (29.4% in HIV-infected individuals) [21], and Brazil (20.5% in HIV-infected individuals) [24]. A systematic review among the general population in countries of the European Union and European Economic Area and the United Kingdom estimated a prevalence of HBV of 0.5% from 2018 to 2021, which was lower than the 0.7% obtained up to 2017 [25]. Higher prevalence rates were estimated in the Western Pacific region (7.1%) and the African region (6.5%) [22].

In Egypt, the prevalence of HBV was estimated at 3.67% in the period from 2000 to 2022. In subgroup analysis among high-risk populations, the prevalence was 5.86%, with the highest prevalence observed in patients with chronic liver disease (34%) and cancer patients (25.5%) [26]. There was only one study reporting the prevalence of HBV in HIV-infected individuals, and it was 6.4% [27]. This data was close to that found in the present study, whereas the prevalence in HIV-infected individuals was estimated at 5.0%. Among them, the highest prevalences were observed in Mexico (29.4%) [21], Brazil (20.5%) [24], and Jamaica (10.9%) [28]. HBV and HIV share the same routes of transmission, which explains the occurrence of coinfection [29].

In LAC, the prevalence of HBV in HIV-infected individuals was previously estimated at 7.0% by 2016 [30]. Globally, systematic reviews on HBV-HIV coinfection have estimated prevalence rates ranging from 7.6% to 8.4%, being higher in the West and Central Africa region (12.4% to 16.4%) [31, 32] and among people who inject drugs (11.8%) [31]. Leumi et al. [32] found a prevalence of coinfection of 5.1% in LAC, almost the same as the estimate in our study. These data are a warning, as in this coinfection, the liver disease may progress faster. Furthermore, HIV-infected individuals showed a lower response to HBV vaccination, and double-dose vaccines or with novel adjuvants such as Heplisav-B may improve seroprotection rates [29]. A study among 6,925 HIV-infected adults who were candidates for hepatitis B vaccination found that only 9% of them received the 3-dose vaccine within 1 year [33].

Established coinfections with HBV, particularly with HIV and hepatitis C virus (HCV), further accelerate disease progression, heighten the risk of hepatocellular carcinoma, and complicate antiviral management [29, 34]. Additionally, SARS-CoV-2 induces prolonged immune dysregulation and autoimmune activation that drive long COVID [35, 36]. Long COVID may cause multi-organ damage, including the liver [35], and autoimmune disorders such as myasthenia gravis have been reported following infections with HBV, HCV, HIV, and other viruses [36]. Surveillance, treatment, and prevention strategies should therefore consider overlapping infections involving HBV.

The American Association for the Study of Liver Diseases recommends groups that should be screened primarily for HBV infection, and these include HIV-infected individuals, indigenous populations of Greenland and Northern Canada, among others [12]. There are likely few data from indigenous populations in LAC. In the present systematic review, we focused on studies from LAC, and the highest prevalence of HBV was estimated at 6.0% among indigenous people. Among them, the highest prevalences were observed in Venezuela (29.5% and 14.3%) [23, 37], Mexico (17.5%) [21], and Brazil (9.6%) [38]. Vasconcelos et al. [39] recently discussed that HBV, along with hepatitis C virus and hepatitis D virus, was widely distributed among some indigenous communities, and, at the same time, the prevalence of vaccine-induced immunity to hepatitis B was still low (20.5%).

In accordance with our finding, a systematic review in Latin America also described high prevalences of HBV in indigenous people between 2000 and 2016, highlighting Venezuela and Mexico with the highest prevalences. The authors warn of gaps in existing data on the prevalence of HBV and other infectious diseases in indigenous people, and highlight the need for an intercultural approach between health and service delivery to this population [40]. Interestingly, the meta-analyses we found were among indigenous Australians (Aboriginal). From 2000 to 2011, the estimated prevalence of HBV was four times higher among indigenous (3.96%) compared to non-indigenous (0.90%) people [41]. In an update from 2000 to 2018, the prevalence did not change much, being 3.5% among indigenous Australians [42].

We observed a decrease in the prevalence of HBV from 2.0% in the period of 2000–2008 to 1.0% in 2009–2016, and it remains at 1.0% in 2017–2024. A global meta-analysis also found a decrease over time, from 6.0% in 1990 to 4.4% in 2015 and, finally, 4.1% in 2019. Conversely, there was a 5.9% increase in HBV-related death counts between 1990 and 2019. The authors argued that this is a result of population growth, aging, and cohort effects, and that HBV-related death rates have actually decreased [22]. The number of deaths from liver cancer, cirrhosis, and other chronic liver diseases related to HBV in 2019 was 523,003 and was estimated to reach 628,824 by 2030 [43]. There is also the risk of developing extrahepatic manifestations that reduce the quality of life of patients with chronic hepatitis B [44]. In the HIV-infected individuals, we observed an increase in the prevalence of HBV from 6.0% during the period of 2000–2008 to 8.0% in more recent years (2017–2024). The HIV epidemic remains uncontrolled in parts of the world and risk populations, with about 1.3 million new infections in 2024 [45]. Widespread antiretroviral therapy has transformed HIV into a chronic condition, extending survival and ageing the infected population [46]. Consequently, sustaining or increasing prevalence despite declining incidence.

Approximately 13% of the HBV-infected population has been diagnosed [1]. Diagnosis testing of hepatitis B is crucial to identify new patients who should be offered therapeutic approaches, avoiding progression to more advanced stages of liver damage. Furthermore, hepatitis B can be prevented through prophylactic vaccination that can confer long-term immunity, which is necessary for controlling its related complications and HBV spreading [17, 47]. However, Vo-Quang and Lemoine [48] point to the need to develop simplified approaches applicable to constrained-resource regions of the world, as well as to scale up interventions for prevention and control. Another concern is disparities in morbidity, mortality, and outcomes of viral hepatitis due to social determinants of health, which includes social, physical, economic, and political environments [49].

The present systematic review provides accurate and updated data on the prevalence of HBV in LAC using reliable database sources. Furthermore, we analyzed data over a 24-year period, which allowed for a large number of studies and a large sample size. However, there are limitations that should be addressed. We did not identify studies from all countries that are part of LAC, although we found a significant number of studies conducted in the region as a whole. When using meta-analysis, we observed significant heterogeneity between studies, in addition to finding an association between the prevalence of HBV and sample size and year of publication of the studies in the meta-regression analysis. However, in the sensitivity analyses we performed (excluding studies with sample sizes of less than 500 and studies with a high risk of bias), the values found for the prevalence of HBV were very close to the value found in the main meta-analysis (including all studies). Another limitation is that HBsAg-negative results due to surface antigen mutants or occult HBV infection were not captured, possibly leading to underestimation of prevalence. Finally, despite the limitations inherent in the available studies, we believe that the data provided by our review can be added to the existing data in the American continent, providing a better understanding of the distribution of HBV, and allowing the optimization of strategies for its elimination in LAC.

## Conclusion

The present systematic review and meta-analysis on the prevalence of HBV (presence of HBsAg) in LAC found a decrease in the prevalence over time, which has remained in recent years. However, risk subgroups stand out with elevated prevalence, such as indigenous peoples and HIV-infected individuals. These findings highlight the need for regionally tailored public health strategies that address persistent inequities in HBV burden, particularly among indigenous communities. Strengthening vaccination coverage, improving access to diagnosis and care in remote areas, and integrating HBV testing into primary health programs may be relevant to reduce the prevalence of HBV in LAC and contribute to the elimination of this infection.

## Supplementary Information

Below is the link to the electronic supplementary material.

Supplementary Material 1

## Data Availability

The data that support the findings of this study are available in the manuscript and supplementary material.
