# Supplementary material for: Prevalence of hepatitis B in Latin America and the Caribbean: a systematic review and meta-analysis
Source: Arch Virol. 2026 Feb 21;171(3):88. doi: 10.1007/s00705-026-06562-z (PMC12924836; doi:10.1007/s00705-026-06562-z)
Supplement: Supplementary file 1 — Supplementary Material 1 [file 705_2026_6562_MOESM1_ESM.docx]

**Prevalence of hepatitis B in Latin America and the Caribbean: a systematic review and meta-analysis**

**Archives of Virology**

Mariana Cavalheiro Magri^1,2^*, Caroline Manchiero^1,2^, Bianca Peixoto Dantas^1,2^, Wanderley Marques Bernardo^3^, Edson Abdala^1,2,4^, Fátima Mitiko Tengan^1,4^

^1^Laboratorio de Investigacao Medica em Hepatologia por Virus (LIM-47), Hospital das Clinicas HCFMUSP, Faculdade de Medicina, Universidade de Sao Paulo, Sao Paulo, Brazil

^2^Instituto de Medicina Tropical de Sao Paulo, Faculdade de Medicina, Universidade de Sao Paulo, Sao Paulo, Brazil

^3^Faculdade de Medicina, Universidade de Sao Paulo, Sao Paulo, Brazil

^4^Departamento de Infectologia e Medicina Tropical, Faculdade de Medicina, Universidade de Sao Paulo, Sao Paulo, Brazil

***Corresponding author:** E-mail: mariana.magri@hc.fm.usp.br

**Supplementary Material**

**Supplementa****ry Material S1.** Literature search strategies for articles on the prevalence of HBV in Latin America and the Caribbean.

**1. Medline through the Pubmed platform**

("Hepatitis B"[Mesh] OR "Hepatitis B, Chronic"[Mesh] OR "Hepatitis B virus"[Mesh] OR "Hepatitis B Surface Antigens"[Mesh] OR "Hepatitis B e Antigens"[Mesh]) AND ("Prevalence"[Mesh] OR "Epidemiology"[Mesh] OR "epidemiology" [Subheading] OR "Cross-Sectional Studies"[Mesh]) AND (argentina OR bolivia OR brazil OR chile OR colombia OR costa rica OR cuba OR dominican republic OR ecuador OR el salvador OR guatemala OR haiti OR honduras OR mexico OR nicaragua OR panama OR paraguay OR peru OR puerto rico OR uruguay OR venezuela OR "Latin America" OR aruba OR "ANTIGUA AND BARBUDA" OR aruba OR bahamas OR barbados OR bonaire OR british virgin islands OR cayman islands OR curacao OR dominica OR grenada OR guadalupe OR jamaica OR martinique OR montserrat OR saba OR saint barthelemy OR "SAINT KITTS AND NEVIS" OR saint lucia OR saint martin OR "SAINT VINCENT AND THE GRENADINES" OR sint eustatius OR sint maarten OR "TRINIDAD AND TOBAGO" OR "TURKS AND CAICOS ISLANDS" OR united states virgin islands OR belize OR french guiana OR guyana OR suriname OR caribbean) NOT ( "Therapeutics"[Mesh] OR "therapy" [Subheading] )

Filters applied: from 01/01/2000 to 18/06/2024

**2. Web of science**

Hepatitis b AND prevalence AND Latin America or Caribbean Not treatment

Filters applied: from 2000-01-01 to 2024-06-18=59

**3. LILACS**

('hepatite b' OR hbv OR 'hepatitis b') AND (prevalencia OR prevalence) AND ('latin america' OR caribbean)

**4. Embase**

'hepatitis b':ab,ti AND (south:ab,ti AND 'central america':ab,ti OR caribbean:ab,ti) AND prevalence:ab,ti AND [2000-2024]/py

**Supplementary Material S2.** Characteristics of the studies included in the systematic review and meta-analysis on the prevalence of HBV in Latin America and the Caribbean.

| **Author** | **Year** | **Sampling period** | **Country** | **Study population** | **Sample** | **Male** | **Age** | **Positive HBsAg** | **Quality** |
| --- | --- | --- | --- | --- | --- | --- | --- | --- | --- |
|  |  |  |  |  | **size** | **(%)** |  |  | **score** |
| Astorga MJ | 2024 | 2018-2019 | Chile | Pregnant women | 1355 | 0 | 27.5^a^ | 4 | 5 |
| Santos APA | 2024 | 2012-2018 | Brazil | Blood donors | 1142228 |  |  | 952 | 5 |
| Vasconcelos MPA | 2024 | 2015 | Brazil | Indigenous people | 430 | 41.9 | 26^b^ | 38 | 6 |
| Motta-Castro ARC | 2023 | 2016 | Brazil | MSM | 3178 | 100 |  | 34 | 6 |
| Marques JMS | 2023 | 2017-2020 | Brazil | Inmates | 1083 | 85.8 | 30^b^ | 9 | 8 |
| de Gois JG | 2022 | 2015-2016 | Brazil | Inmates | 1132 | 100 |  | 7 | 7 |
| Lafer MM | 2022 | 2018-2020 | Brazil | Indigenous people | 81 | 51 | 28^a^ | 0 | 5 |
| Laguna-Meraz S | 2022 | 2016-2017 | Mexico | General population | 7894 |  |  | 80 | 5 |
| Villar LM | 2022 | 2013-2017 | Brazil | Chronic kidney disease | 644 | 53.7 | 50.3^a^ | 38 | 5 |
| Albán Fernandez L | 2021 | 2018 | Peru | General population | 404 | 30.7 | 46.2^a^ | 5 | 5 |
| MacDonald-Ottevanger MS | 2021 | 2012 | Surinam | General population | 1988 | 53 | 38.7^b^ | 63 | 5 |
| Miranda NTGP | 2021 | 2005-2006 | Brazil | Sex workers | 365 | 0 | 27.4^a^ | 11 | 5 |
| Nunes JDC | 2021 | 2012-2016 | Brazil | General population | 3983 | 42.2 |  | 92 | 6 |
| Silva RJS | 2021 | 2013-2018 | Brazil | Illicit drug users | 1074 | 65.6 |  | 50 | 5 |
| Sánchez-Vanegas G | 2021 | 2019 | Colombia | Inmates | 447 | 100 | 33^b^ | 2 | 6 |
| Ferreira-Junior PA | 2020 | 2013 | Brazil | General population | 623 | 67.6 | 30.8^a^ | 47 | 6 |
| Cabezas C | 2020 | 2012 | Peru | Indigenous people | 963 | 47.9 | 23^a^ | 32 | 7 |
| Cabezas C | 2020 | 2014-2015 | Peru | General population | 5183 | 28.5 |  | 20 | 8 |
| Calux SJ | 2020 | 2013-2014 | Brazil | HIV | 232 |  |  | 7 | 4 |
| do Nascimento CT | 2020 | 2017-2018 | Brazil | Inmates | 37497 |  |  | 16 | 4 |
| Hernández-Romano P | 2020 | 2014-2017 | Mexico | Blood donors | 28016 |  |  | 29 | 5 |
| Patzi-Churqui M | 2020 | 2015-2019 | Bolivia | Rural population | 389 | 0 |  | 6 | 5 |
| Piauiense JNF | 2020 | 2015 | Brazil | Intravenous drug user | 308 | 63.3 |  | 7 | 5 |
| Rezende GR | 2020 | 2013-2014 | Brazil | Inmates | 3368 | 84.6 |  | 18 | 7 |
| Vargas L | 2020 | 2016-2017 | Brazil | Pregnant women | 2099 | 0 | 27.3^a^ | 8 | 8 |
| Villar LM | 2020 | 2014 | Argentina | General population | 622 | 44.6 | 36.6^a^ | 11 | 5 |
| Weitzel T | 2020 | 2014-2015 | Chile | HIV | 399 | 93 | 38.3^a^ | 23 | 7 |
| Mendizabal M | 2020 | 2018-2020 | Argentina | Inmates | 1141 | 73.5 | 31.1^a^ | 7 | 7 |
| Benedetti MSG | 2020 | 2017 | Brazil | Inmates | 168 | 0 | 36.5^a^ | 0 | 8 |
| Douine M | 2019 | 2015 | French Guiana | Miners | 416 |  |  | 19 | 5 |
| Frade PC | 2019 | 2015-2017 | Brazil | Sex workers | 153 | 0 | 23.5^a^ | 4 | 4 |
| Guimarães LCC | 2019 | 2016 | Brazil | General population | 378 | 49.7 | 31^b^ | 3 | 6 |
| Puga MAM | 2019 | 2014-2017 | Brazil | Inmates with tuberculosis | 279 | 100 | 29^b^ | 4 | 6 |
| da Silva BEB | 2019 | 2014-2017 | Brazil | HIV | 435 | 0 | 38^b^ | 9 | 6 |
| Blanco RY | 2018 | 2011 | Venezuela | Indigenous people | 548 | 52 | 27^b^ | 10 | 5 |
| Cavaretto L | 2018 | 2011-2014 | Brazil | Manicurists | 514 | 0.4 | 34.7^a^ | 2 | 6 |
| de Castro Rocha DFN | 2018 | 2016 | Brazil | Sugarcane cutters | 937 | 100 | 35.4^a^ | 7 | 7 |
| Klein G | 2018 | 2015 | Brazil | Recyclable waste collectors | 73 | 12.3 | 33.7^a^ | 9 | 5 |
| Ramírez-Soto MC | 2018 | 2010-2015 | Peru | Blood donors | 2895 | 63.4 | 30.7^a^ | 33 | 5 |
| Villar LM | 2018 | 2011-2017 | Brazil | General population | 948 | 46.7 | 27.8^a^ | 4 | 5 |
| Villarroel-Torrico M | 2018 | 2013 | Bolivia | Inmates | 219 | 0 | 35.9^a^ | 1 | 5 |
| Abel S | 2018 | 2014 | Martinica | Inmates | 461 |  |  | 4 | 4 |
| Alvarez L | 2017 | 2010-2012 | Peru | Blood donors | 13887 |  |  | 77 | 5 |
| Andrade AP | 2017 | 2013-2015 | Brazil | Illicit drug users | 466 | 68.3 | 28.4^a^ | 18 | 5 |
| Belaunzaran-Zamudio PF | 2017 | 2011-2012 | Mexico | Inmates | 3182 |  |  | 13 | 7 |
| Bórquez C | 2017 | 2013 | Chile | Inmates | 140 | 100 | 36.7^a^ | 4 | 7 |
| Jose-Abrego A | 2017 | 2012-2015 | Mexico | Chronic liver disease | 272 |  |  | 2 | 7 |
| Jose-Abrego A | 2017 | 2012-2015 | Mexico | HIV | 228 |  |  | 67 | 7 |
| Jose-Abrego A | 2017 | 2012-2015 | Mexico | Indigenous people | 57 |  |  | 10 | 7 |
| Matos MA | 2017 | 2009-2010 | Brazil | Sex workers | 402 | 0 |  | 6 | 6 |
| Morales J | 2017 | 2012-2015 | Peru | Blood donors | 28263 |  |  | 156 | 4 |
| Pinheiro RS | 2017 | 2012-2013 | Brazil | Non-intravenous drug user | 93 | 0 | 17^b^ | 1 | 4 |
| Ribeiro Barbosa J | 2017 | 2014-2015 | Brazil | HIV | 154 | 85 |  | 6 | 5 |
| Ribeiro Barbosa J | 2017 | 2014-2015 | Brazil | Chronic kidney disease | 143 | 68.5 |  | 10 | 5 |
| Ribeiro Barbosa J | 2017 | 2014-2015 | Brazil | Coagulopathy | 51 | 98 |  | 2 | 5 |
| Santos MB | 2017 | 2010-2011 | Brazil | Schistosoma mansoni | 170 |  |  | 3 | 5 |
| da Silva EF | 2016 | 2010-2013 | Brazil | Chronic hepatitis C | 1000 | 51.4 |  | 8 | 5 |
| Iglecias LM | 2016 | 2014-2015 | Brazil | Inmates with tuberculosis | 216 | 95.4 | 32.8^a^ | 3 | 6 |
| Oliveira MP | 2016 | 2014 | Brazil | MSM | 522 | 100 |  | 5 | 5 |
| Pisano MB | 2016 | 2011-2014 | Argentina | Blood donors | 70102 |  |  | 36 | 4 |
| Bottecchia M | 2015 |  | Brazil | Civil construction | 1200 |  |  | 8 | 3 |
| Brandão NA | 2015 | 2011 | Brazil | HIV | 495 | 73.9 | 40^b^ | 19 | 4 |
| di Filippo D | 2015 | 2011-2013 | Colombia | Indigenous people | 862 |  |  | 23 | 4 |
| Lopez-Balderas N | 2015 | 2006-2010 | Mexico | Blood donors | 56377 | 89 |  | 61 | 4 |
| Melo LVL | 2015 | 2011-13 | Brazil | Urban and rural population | 1001 | 39.4 | 46.7^a^ | 1 | 5 |
| Moraes TC | 2015 | 2011-2012 | Brazil | Mental illness | 333 | 36.3 |  | 3 | 4 |
| Pacher BM | 2015 | 2013 | Brazil | Deafness | 88 | 56.8 | 35^b^ | 2 | 4 |
| Pinto FP | 2015 | 2013 | Brazil | General population | 2936 | 31 | 31^b^ | 4 | 4 |
| Villar LM | 2015 | 2013 | Brazil | Military | 433 | 100 |  | 0 | 4 |
| Bautista-Arredondo S | 2015 | 2010 | Mexico | Inmates | 17084 | 89.9 |  | 26 | 6 |
| Araujo MP | 2014 | 2011 | Brazil | Athletes | 50 | 0 | 20^a^ | 0 | 3 |
| Boa-Sorte N | 2014 | 2009-2010 | Brazil | Pregnant women | 692 | 0 | 27.1^a^ | 2 | 4 |
| Carmo RA | 2014 | 2006 | Brazil | Mental illness | 2206 | 48 |  | 45 | 5 |
| Ciaccia MC | 2014 | 2007 | Brazil | Children and teenagers | 4680 |  |  | 4 | 4 |
| Delfino CM | 2014 | 2003-2009 | Argentina | Blood donors | 56983 |  |  | 109 | 4 |
| Fernandes CN | 2014 | 2005-2009 | Brazil | Pregnant women | 1616 | 0 |  | 10 | 4 |
| Flichman DM | 2014 | 2004-2011 | Argentina | Blood donors | 530.383 |  |  | 1050 | 4 |
| Freitas SZ | 2014 | 2009-2011 | Brazil | HIV | 848 | 57 | 41.6^a^ | 21 | 5 |
| Gelu-Simeon M | 2014 | 2006-2007 | Guadeloupe | General population | 2200 | 40.3 | 43^b^ | 31 | 5 |
| Jaspe RC | 2014 | 2002-2011 | Venezuela | HIV | 418 | 64 |  | 13 | 5 |
| Marinho TA | 2014 | 2010-2011 | Brazil | Recyclable waste collectors | 431 | 37.6 | 36.9^a^ | 3 | 5 |
| Martins S | 2014 | 2012-2013 | Brazil | HIV | 300 | 59.7 | 44.6^a^ | 7 | 5 |
| Raboni SM | 2014 | 2011-2013 | Brazil | HIV | 88 |  |  | 18 | 4 |
| Soares CC | 2014 | 2005-2006 | Brazil | MSM | 558 | 100 | 23^b^ | 19 | 4 |
| Barros LA | 2013 | 2007-2008 | Brazil | Inmates | 148 | 0 |  | 1 | 4 |
| Ferezin RI | 2013 | 2010 | Brazil | Pregnant women | 1483 | 0 |  | 8 | 4 |
| Johnston LG | 2013 | 2008 | Dominican Republic | Gays, trans and MSM | 1388 |  |  | 31 | 4 |
| Maccarini J | 2013 | 2009-2010 | Brazil | Blood donors | 3180 | 59.4 | 27.2^a^ | 2 | 4 |
| Menegol D | 2013 | 2008-2011 | Brazil | General population | 31749 |  |  | 519 | 5 |
| Moreira M | 2013 |  | Brazil | HIV | 200 | 54.5 | 39^a^ | 1 | 4 |
| Moreira M | 2013 |  | Brazil | HTLV-1 | 213 | 46.6 | 49^a^ | 5 | 4 |
| Schuelter-Trevisol F | 2013 | 2009 | Brazil | Sex workers | 147 | 8.8 | 28^a^ | 5 | 4 |
| Távora LG | 2013 | 2008-2010 | Brazil | HIV | 671 |  |  | 25 | 4 |
| Aires RS | 2012 | 2008-2010 | Brazil | Tuberculosis | 402 | 71.9 | 44.1^a^ | 13 | 5 |
| Castilho M da C | 2012 | 2008 | Brazil | General population | 225 | 53.8 | 21.3^a^ | 23 | 5 |
| Contrera-Moreno L | 2012 | 2010 | Brazil | Firemen | 308 | 89.9 | 36.4^a^ | 3 | 5 |
| de Almeida MK | 2012 | 2010-2011 | Brazil | Rural population | 668 | 39 | 38^a^ | 13 | 5 |
| Delfino CM | 2012 | 2007-2008 | Argentina | Indigenous people | 561 | 36 | 33.5^a^ | 6 | 5 |
| Ormaeche M | 2012 | 2007-2008 | Peru | Pregnant women | 552 | 0 |  | 11 | 5 |
| Ormaeche M | 2012 | 2007-2008 | Peru | Pregnant women male partners | 377 | 100 |  | 15 | 5 |
| Ramírez-Soto MC | 2012 | 2000-2009 | Peru | Blood donors | 3445 | 56 |  | 66 | 4 |
| Souza MT | 2012 | 2009 | Brazil | Pregnant women | 541 | 0 | 24^a^ | 5 | 6 |
| Alvarado-Mora MV | 2011 |  | Colombia | General population | 618 | 26.4 |  | 35 | 5 |
| Cardona NE | 2011 | 2002-2004/2009 | Venezuela | Indigenous people | 150 |  |  | 2 | 3 |
| dos Ramos Farias MS | 2011 | 2006-2009 | Argentina | Sex workers | 273 | 100 | 29^b^ | 5 | 5 |
| dos Ramos Farias MS | 2011 | 2006-2009 | Argentina | Sex workers | 114 | 100 | 27^b^ | 1 | 5 |
| Juárez-Figueroa LA | 2011 | 2004 | Mexico | General population | 295 | 36.9 | 37.3^a^ | 4 | 5 |
| Livramento A | 2011 | 2007-2008 | Brazil | Children and teenagers | 393 | 46.5 | 12.5^a^ | 3 | 5 |
| Ramírez-Soto MC | 2011 | 2010 | Peru | University students | 240 | 40 | 24.1^a^ | 6 | 4 |
| Scaraveli NG | 2011 | 2008 | Brazil | Teenagers | 418 | 39 | 13.7^a^ | 1 | 4 |
| Silva JLA | 2011 | 2008 | Brazil | Schistosoma mansoni | 230 | 41.3 | 55^a^ | 7 | 5 |
| Tonial GC | 2011 | 2008 | Brazil | Teenagers | 353 | 36.5 | 13.3^a^ | 4 | 6 |
| Duarte MC | 2010 | 2002-2004 | Venezuela | Indigenous people | 414 |  |  | 21 | 5 |
| Duarte MC | 2010 | 2002-2004 | Venezuela | Indigenous people | 231 |  |  | 33 | 5 |
| Lama JR | 2010 | 2002-2003 | Peru | MSM | 2703 | 100 | 24^b^ | 87 | 4 |
| Laufer N | 2010 | 2004-2005 | Argentina | HIV | 593 | 65.6 |  | 22 | 4 |
| Machado Filho AC | 2010 | 2008 | Brazil | Pregnant women | 674 | 0 | 23.9^a^ | 5 | 5 |
| Mahamat A | 2010 | 2007 | French Guiana | Pregnant women | 2347 | 0 | 27.4^a^ | 36 | 5 |
| Roman S | 2010 | 2005 | Mexico | Indigenous people | 147 | 30 | 29^a^ | 2 | 5 |
| Roman S | 2010 | 2003-2005 | Mexico | Indigenous people | 159 | 28 | 41^a^ | 15 | 5 |
| Stief AC | 2010 |  | Brazil | Inmates | 408 | 40.7 | 26^a^ | 2 | 5 |
| Voigt AR | 2010 | 2007-2008 | Brazil | Children and teenagers | 384 | 42.4 | 12.6^a^ | 0 | 6 |
| Calderón GM | 2009 |  | Mexico | Polytransfused | 300 | 59 | 31^a^ | 21 | 4 |
| Ferreira RC | 2009 | 2005-2006 | Brazil | Non-intravenous drug user | 852 | 91.4 | 27.5^a^ | 9 | 9 |
| Guimarães MD | 2009 |  | Brazil | Mental illness | 2238 | 48.1 |  | 37 | 5 |
| Lima LH | 2009 | 1999 | Brazil | Postpartum | 330 | 0 |  | 4 | 7 |
| Lima LH | 2009 | 1999 | Brazil | Pregnant women | 197 | 0 |  | 2 | 7 |
| Matos MA | 2009 | 2004 | Brazil | General population | 878 | 0 | 28.3^a^ | 16 | 7 |
| Pérez CC | 2009 | 1990-2007 | Chile | HIV | 311 | 92.6 |  | 19 | 4 |
| Valerio-Ureña J | 2009 | 2005 | Mexico | Blood donors | 8650 | 93 | 32.6^a^ | 5 | 6 |
| Almeida D | 2008 | 1999-2003 | Brazil | Rural population | 1476 |  |  | 38 | 4 |
| Aquino JA | 2008 | 2002-2005 | Brazil | General population | 11282 |  |  | 410 | 4 |
| Chacaltana A | 2008 | 2007 | Peru | Military | 3343 | 93.2 | 38.9^a^ | 8 | 5 |
| Matos MA | 2008 | 2005-2006 | Brazil | Truck drivers | 641 | 99.2 | 40.6^a^ | 16 | 7 |
| Miranda AE | 2008 | 2006 | Brazil | General population | 1029 | 0 | 23^b^ | 9 | 5 |
| Monsalve-Castillo F | 2008 |  | Venezuela | Indigenous people | 149 | 60.4 |  | 44 | 4 |
| Motta-Castro AR | 2008 |  | Brazil | Afrodescendant | 239 |  |  | 20 | 4 |
| Nascimento MC | 2008 | 2003 | Brazil | Blood donors | 3398 |  |  | 12 | 4 |
| Rojas-Garcia M | 2008 | 2005 | Mexico | Blood donors | 6647 | 92.68 | 31.73^a^ | 1 | 4 |
| Tolentino YF | 2008 | 2002-2004 | Brazil | Inflammatory bowel disease | 176 | 38.6 |  | 4 | 5 |
| Trenchi A | 2007 | 2000 | Argentina | Pregnant women | 2705 |  |  | 7 | 4 |
| Trenchi A | 2007 | 2000 | Argentina | Blood donors | 16002 |  |  | 4 | 4 |
| Zago AM | 2007 | 1993-2004 | Brazil | HIV | 851 |  |  | 32 | 5 |
| Almeida D | 2006 | 1999 | Brazil | Rural population | 1476 |  |  | 38 | 5 |
| Andrade AF | 2006 | 1998-2005 | Brazil | Blood donors | 128497 |  |  | 347 | 4 |
| Batista SM | 2006 | 2003-2004 | Brazil | Health professionals | 474 | 36.3 | 38.5^a^ | 3 | 5 |
| Bellíssimo-Rodrigues WT | 2006 | 2001-2002 | Brazil | Health professionals | 135 | 37 | 34.5^a^ | 1 | 4 |
| Bertolini DA | 2006 | 1998-2002 | Brazil | Pregnant women | 3188 | 0 | 24.2^a^ | 54 | 4 |
| Braga WS | 2006 | 1998-2003 | Brazil | HIV | 704 | 65.1 |  | 45 | 5 |
| de Almeida Pereira RA | 2006 | 2004 | Brazil | HIV | 1000 | 53 | 37.2^a^ | 37 | 5 |
| Espinoza Holguin M | 2006 |  | Venezuela | Children with cancer | 52 | 59.6 |  | 26 | 4 |
| Ferreira A | 2006 |  | Brazil | Indigenous people | 214 | 37.85 | 29.8^a^ | 0 | 5 |
| Hoyos-Orrego A | 2006 | 2002-2004 | Colombia | HIV | 251 | 85.6 | 37.9^a^ | 2 | 4 |
| Méndez-Sanchez N | 2006 |  | Mexico | Health professionals | 376 | 2.1 | 30.8^a^ | 0 | 4 |
| Tovo CV | 2006 |  | Brazil | HIV | 306 |  |  | 14 | 5 |
| Berra JAP | 2006 | 2003-2004 | Brazil | Inmates | 225 | 0 |  | 3 | 4 |
| Alvarado-Esquievel C | 2005 | 2001-2002 | Mexico | Inmates | 181 | 96 | 32.2^a^ | 5 | 7 |
| de Paula EV | 2005 | 2002-2003 | Brazil | Polytransfused | 353 | 56.7 | 36.1^a^ | 3 | 4 |
| El Khouri M | 2005 | 2000 | Brazil | General population | 267 | 44.4 | 34.2^a^ | 13 | 4 |
| Motta-Castro AR | 2005 | 2002-2003 | Brazil | Afrodescendant | 1058 | 48 | 28.9^a^ | 23 | 5 |
| Remesar M | 2005 | 2002-2004 | Argentina | Polytransfused | 504 | 63.9 | 12^b^ | 1 | 5 |
| Silva PA | 2005 | 2000-2001 | Brazil | Health professionals | 295 |  |  | 2 | 4 |
| Toledo AC | 2005 | 2002 | Brazil | Army recruits | 5820 | 100 | 18.1^a^ | 150 | 7 |
| Valente VB | 2005 | 1996-2001 | Brazil | Blood donors | 25891 | 83.6 |  | 164 | 4 |
| Viana S | 2005 | 2002 | Brazil | General population | 2656 | 42.1 | 32^a^ | 89 | 4 |
| Vinelli E | 2005 | 2002-2003 | Honduras | Polytransfused | 502 |  |  | 53 | 4 |
| Assis SB | 2004 | 1998 | Brazil | Children | 487 | 47.6 | 7.3^a^ | 6 | 6 |
| Monteiro MR | 2004 | 1999-2000 | Brazil | HIV | 406 | 74 | 34.2^a^ | 32 | 4 |
| Souza MG | 2004 | 2022 | Brazil | HIV | 401 | 64.8 |  | 34 | 4 |
| Carreto-Vélez MA | 2003 | 1998-2000 | Mexico | Blood donors | 4010 | 78.9 |  | 45 | 4 |
| Motta-Castro AR | 2003 | 1999-2000 | Brazil | Afrodescendant | 260 | 48.8 | 25^a^ | 24 | 5 |
| Rosini N | 2003 | 1999-2001 | Brazil | Blood donors | 263975 |  |  | 2142 | 4 |
| Russi JC | 2003 | 1999 | Uruguay | Sex workers | 200 | 100 | 30.5^a^ | 6 | 5 |
| Santos EA | 2003 | 1998-2000 | Brazil | HIV | 170 | 80.6 | 39^a^ | 14 | 4 |
| Smikle MF | 2003 | 2001-2002 | Jamaica | HIV | 129 | 38.6 |  | 19 | 5 |
| Vázquez-Martínez JL | 2003 | 2000 | Mexico | Pregnant women | 9992 | 0 | 26^a^ | 99 | 7 |
| Weissenbacher M | 2003 | 2000-2001 | Argentina | Intravenous drug user | 174 | 78.7 |  | 15 | 5 |
| Silva C | 2002 | 1995-1997 | Brazil | Acute hepatitis | 1396 |  |  | 202 | 4 |
| Aguiar JI | 2001 |  | Brazil | Blood donors | 552 |  |  | 4 | 5 |
| Braga WS | 2001 |  | Brazil | Indigenous people | 688 | 51.5 | 22^a^ | 66 | 5 |
| Oliveira LH | 2001 | 1997 | Brazil | Clinic of STI | 440 | 44.8 | 29^a^ | 15 | 4 |
| Rodriguez Lopes CL | 2001 | 1998 | Brazil | Health professionals | 152 | 7.9 | 34.2^a^ | 1 | 4 |
| Smikle MF | 2001 | 1998-1999 | Jamaica | Clinic of STI | 485 | 40.6 | 28^a^ | 15 | 4 |
| Souto FJ | 2001 | 1996 | Brazil | Miners | 520 | 85 | 32^a^ | 37 | 4 |
| Guimarães T | 2001 | 1993-1994 | Brazil | Inmates | 425 | 100 |  | 130 | 7 |
| Mendes-Corrêa MC | 2000 | 1996 | Brazil | HIV | 1693 | 68.2 |  | 96 | 4 |
| Rodríguez L | 2000 |  | Cuba | HIV | 295 | 72.5 | 30^a^ | 15 | 4 |
| Smikle MF | 2000 | 1994-1999 | Jamaica | Non-intravenous drug user | 301 | 91 | 33^a^ | 2 | 4 |
| Catalan-Soares BC | 2000 | 1994 | Brazil | Inmates | 63 | 100 | 30.2^a^ | 11 | 4 |
| Miranda AE | 2000 | 1997 | Brazil | Inmates | 121 | 0 | 30.2^a^ | 9 | 4 |

HBsAg: Hepatitis B surface antigen; HIV: Human immunodeficiency virus; HTLV-1: Human T cell lymphotropic virus type 1; MSM: Men who have sex with men; STI: Sexually transmitted infections. ^a^ Mean age; ^b^ Median age.

**Supplementary Material S3.** Forest plot showing the estimated global prevalence of HBV in Latin America and the Caribbean.


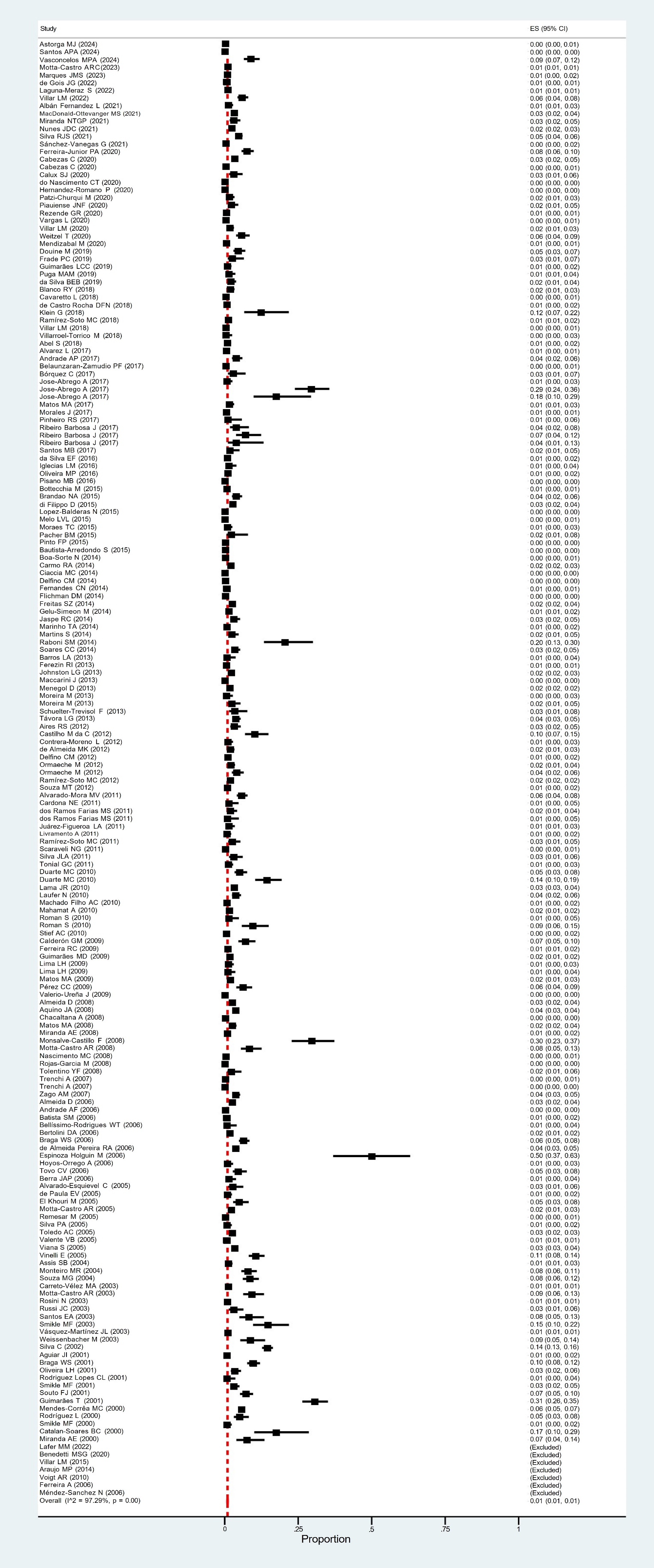


Continuation Supplemental Material S3.


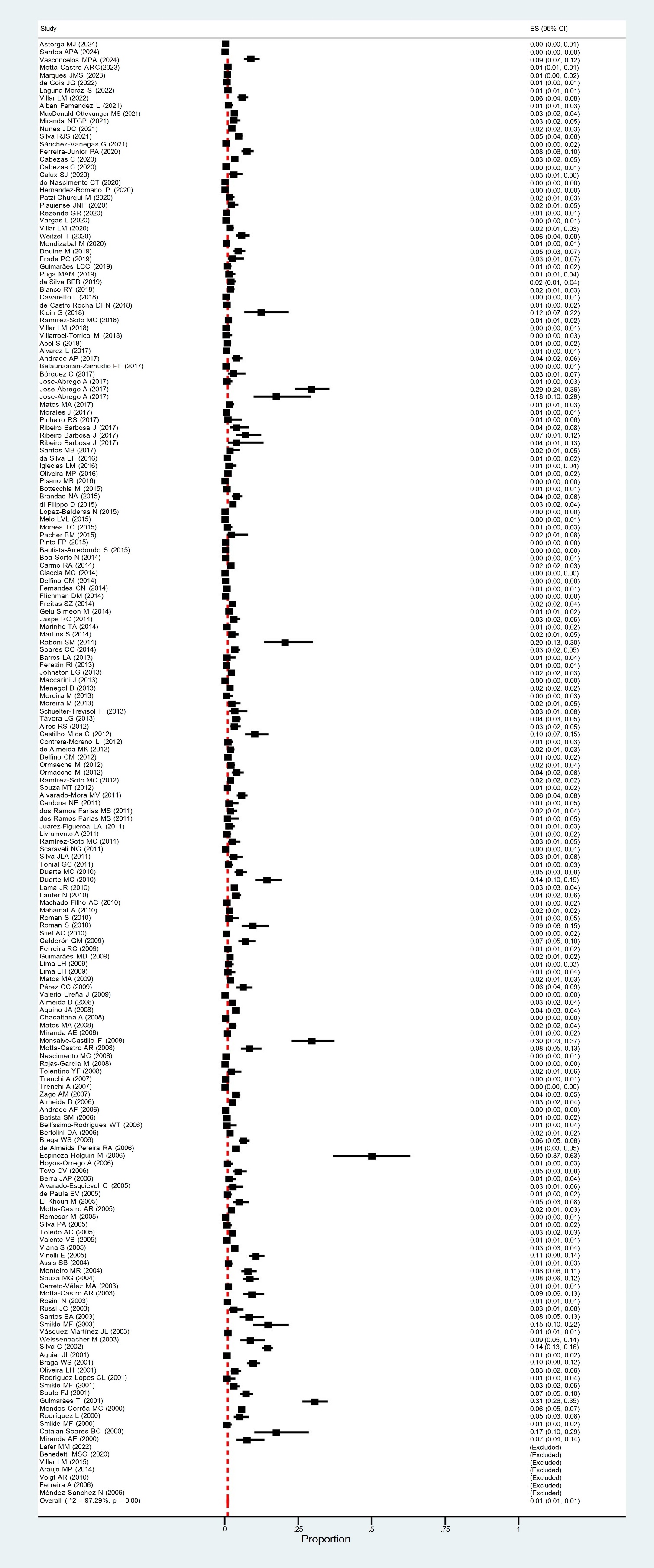


**Supplementary Material S4.** Meta-regression analysis of the HBV prevalence studies in Latin America and the Caribbean.

|  | **Meta-regression coefficient** | **95% CI** | | **p-value** |
| --- | --- | --- | --- | --- |
|  |  | **Low** | **Uper** |  |
| Sample size | -3.10e-06 | -5.07e-06 | -1.14e-06 | 0.002 |
| Publication year | -.0544419 | -.0871482 | -.0217357 | 0.001 |
| Quality score | .0839486 | -.1051688 | .2730661 | 0.384 |

**Supplementary Material S5.** Tests for publication bias of the studies on the prevalence of HBV in Latin America and the Caribbean.

**(A)** Begg`s funnel plot to assess publication bias of the studies on the prevalence of HBV.


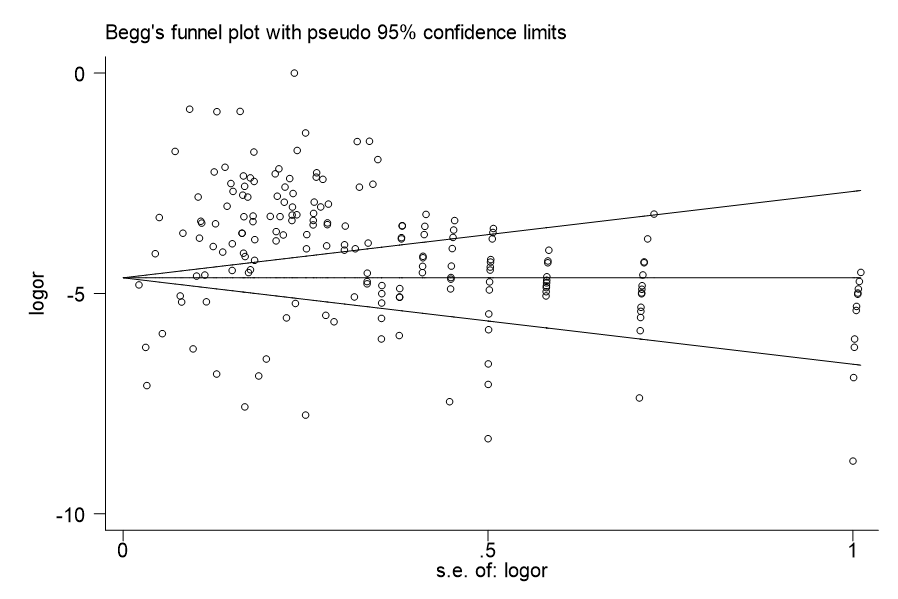


**(B)** Tests for publication bias of the studies on the prevalence of HBV.

**Begg's test**

adj. Kendall's Score (P-Q) = -5676

Std. Dev. of Score = 828.54 (corrected for ties)

Number of Studies = 183

z = -6.85

Pr > |z| = 0.000

z = 6.85 (continuity corrected)

Pr > |z| = 0.000 (continuity corrected)

**Egger's test**

| **Std. Eff** | **Coefficient** | **Std. Err.** | **t** | **95% CI** | | **p-value** |
| --- | --- | --- | --- | --- | --- | --- |
|  |  |  |  | **Low** | **Uper** |  |
| Slope | -5.08053 | .1462661 | -34.73 | -5.369136 | -4.791924 | 0.000 |
| Bias | 4. 695547 | 1.049381 | 4.47 | 2. 624953 | 6.766141 | 0.000 |

**Supplementary Material S6.** Forest plot showing the estimated prevalence of HBV in Latin America and the Caribbean according to sensitivity analyses including studies with **(A)** sample size greater than 200, and **(B)** sample size greater than 500.

**(A)** sample size greater than 200


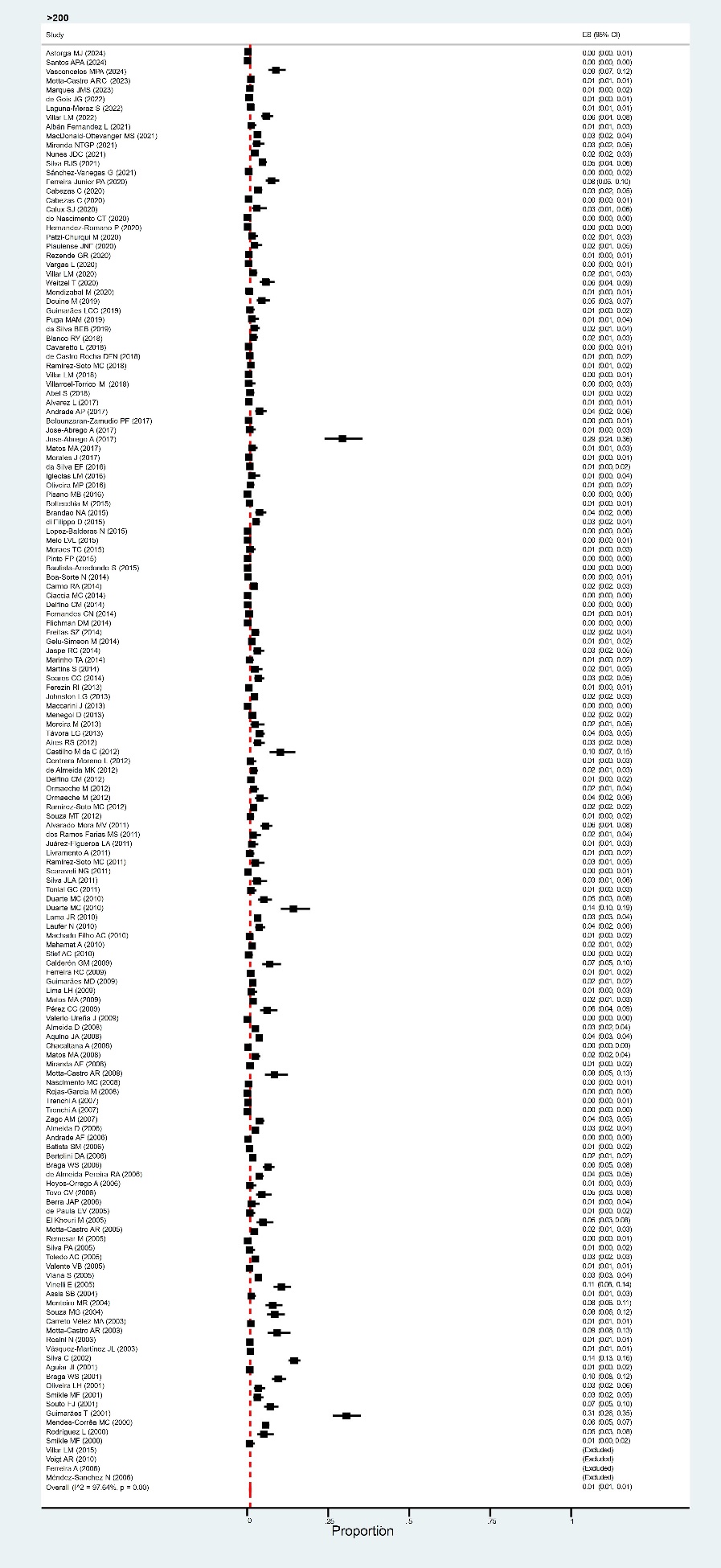


**(B)** sample size greater than 500


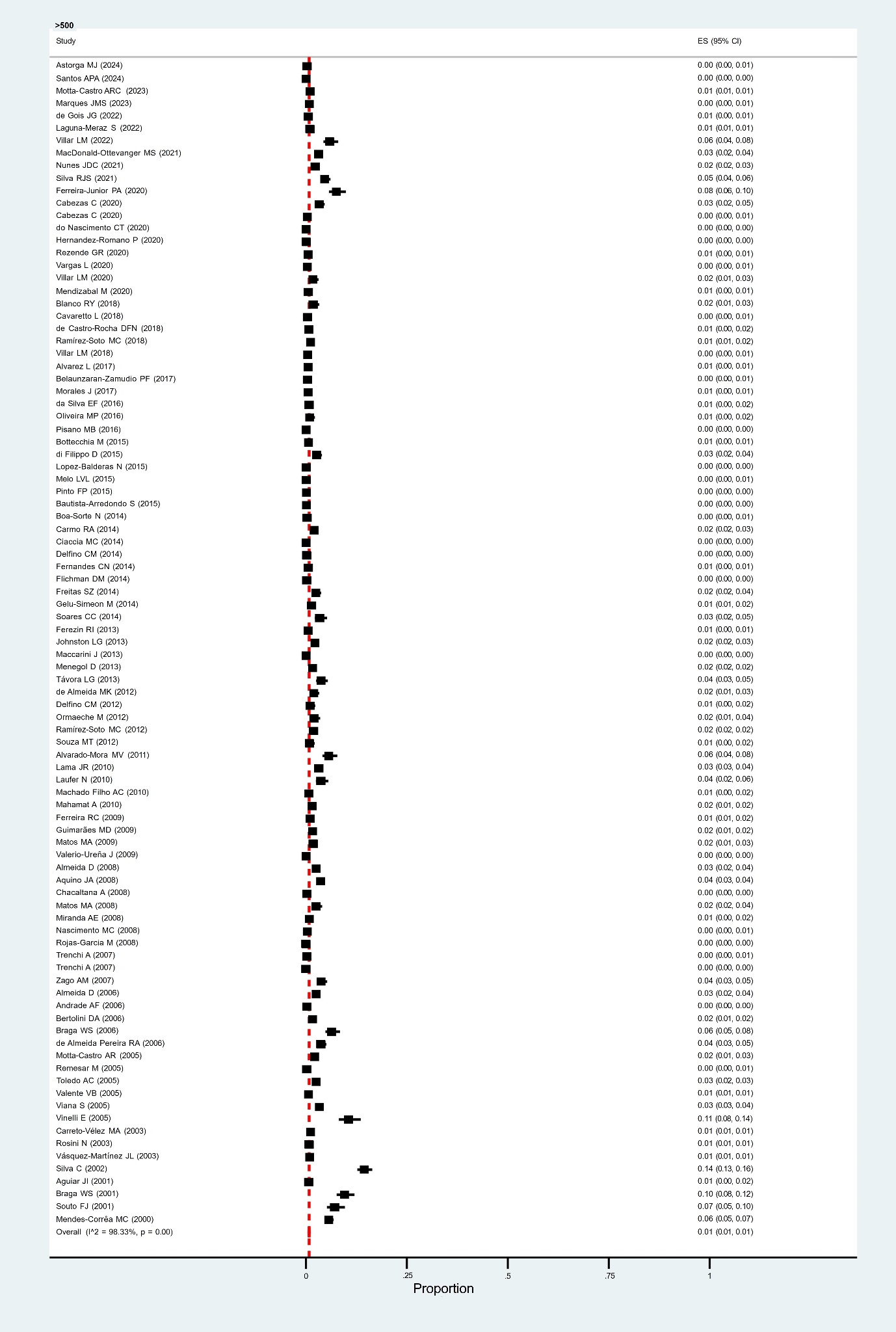


**Supplementary Material S7.** Forest plot showing the estimated prevalence of HBV in Latin America and the Caribbean according to publication period: **(A)** 2000 to 2008, **(B)** 2009 to 2016, and **(C)** 2017 to 2024.

**(A)** 2000 to 2008


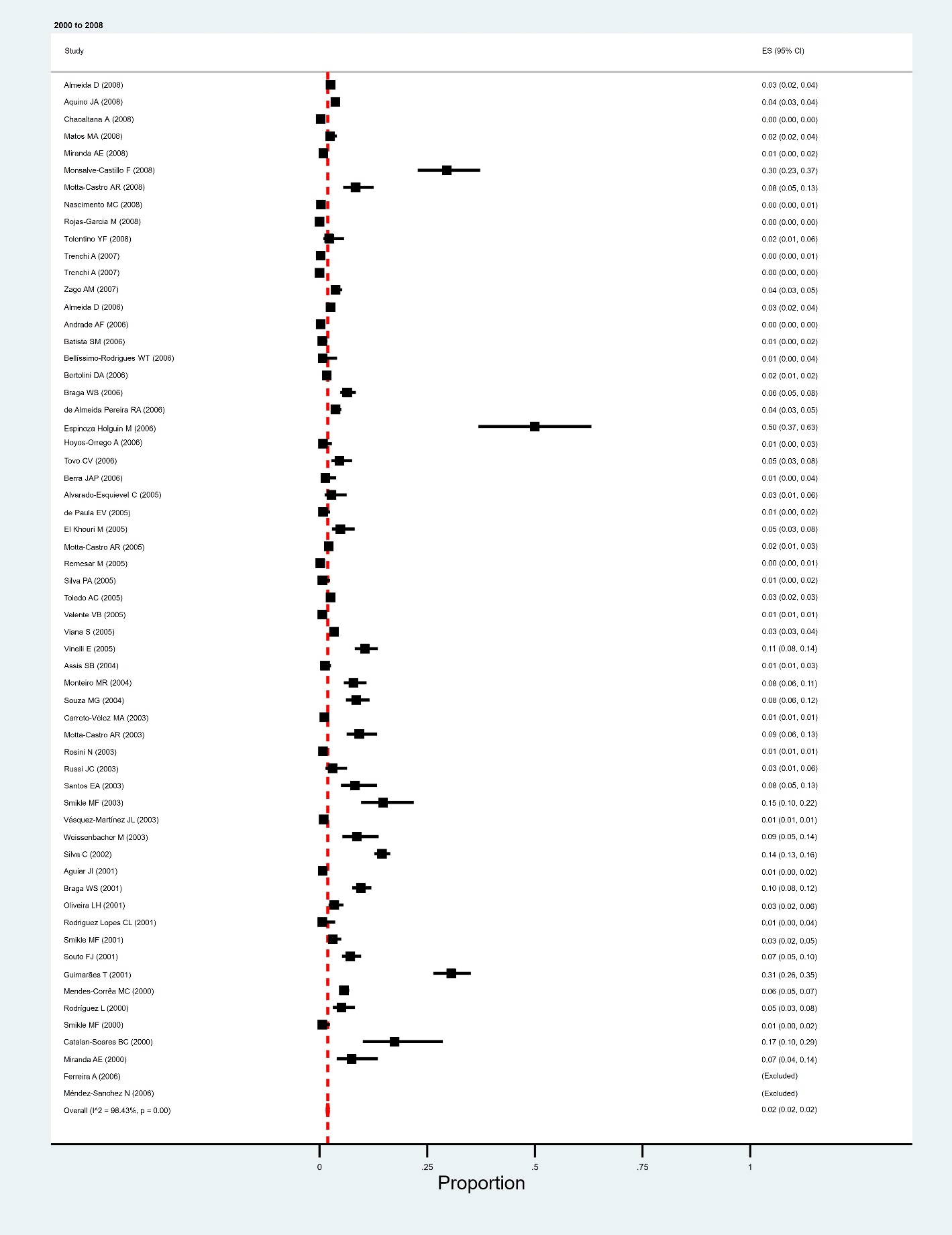


**(B)** 2009 to 2016


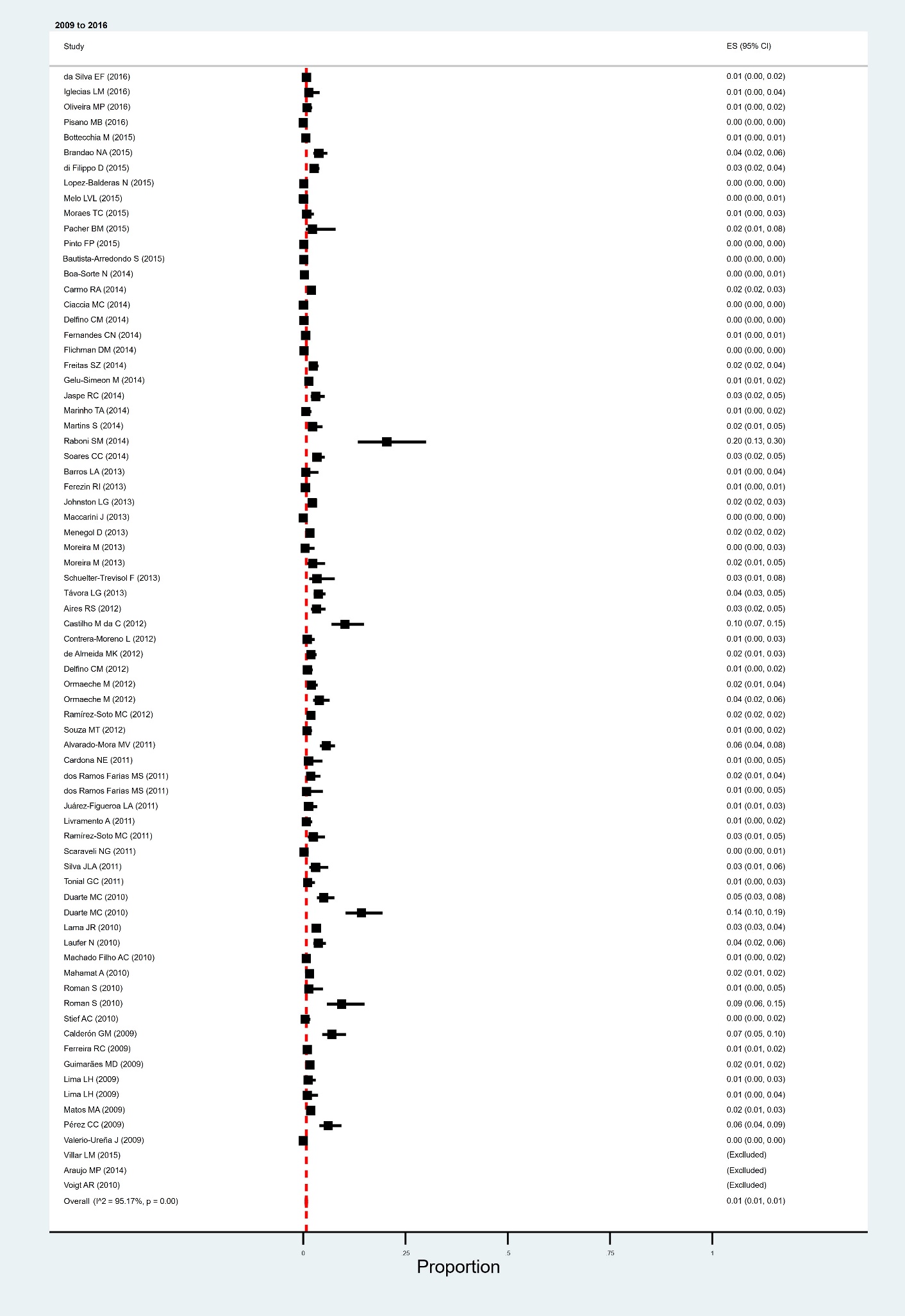


**(C)** 2017 to 2024


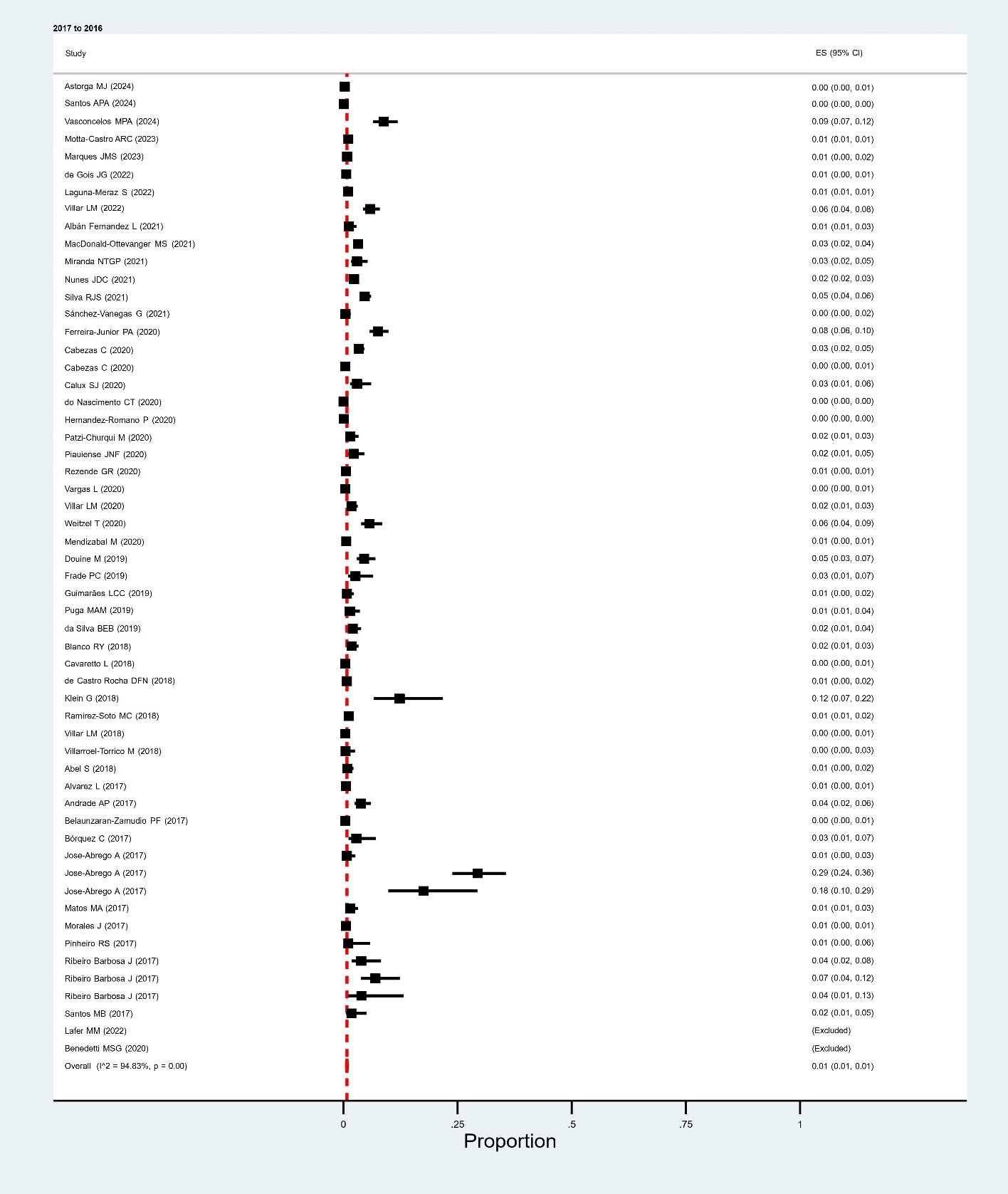


**Supplementary Material S8.** Forest plot showing the estimated prevalence of HBV in the general population of Latin America and the Caribbean, according to publication period: **(A)** 2000 to 2008, **(B)** 2009 to 2016, and **(C)** 2017 to 2024.

**(A)** 2000 to 2008


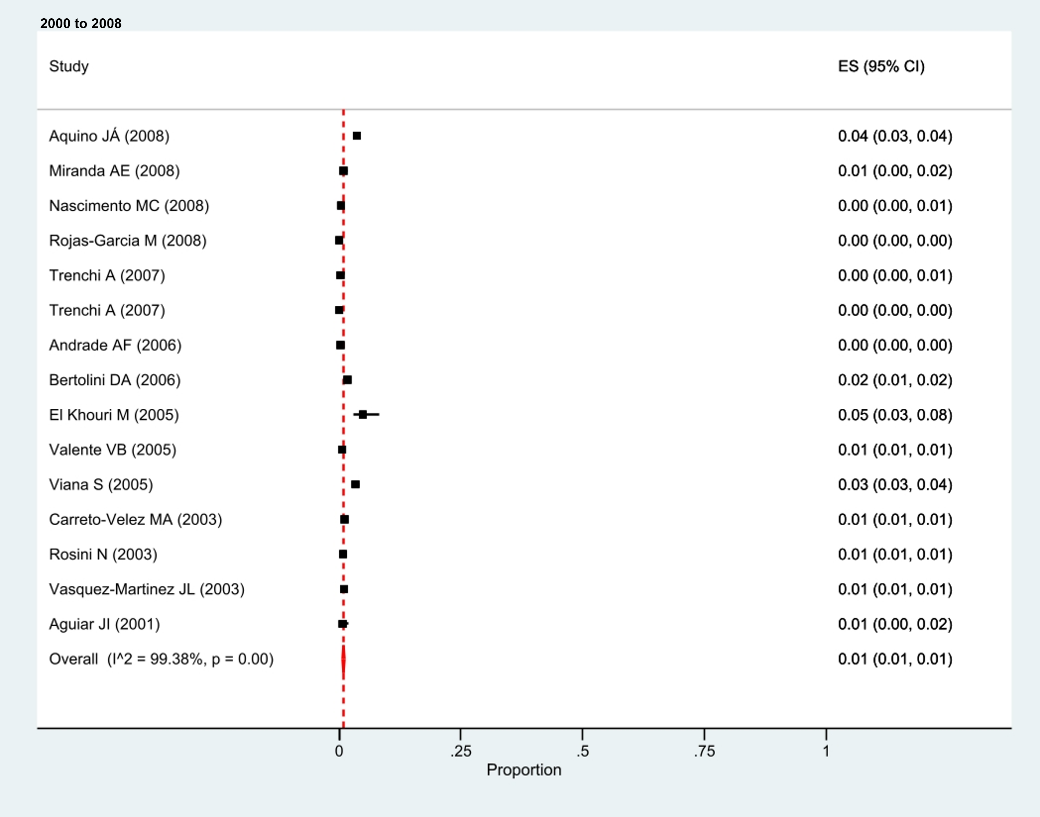


**(B)** 2009 to 2016


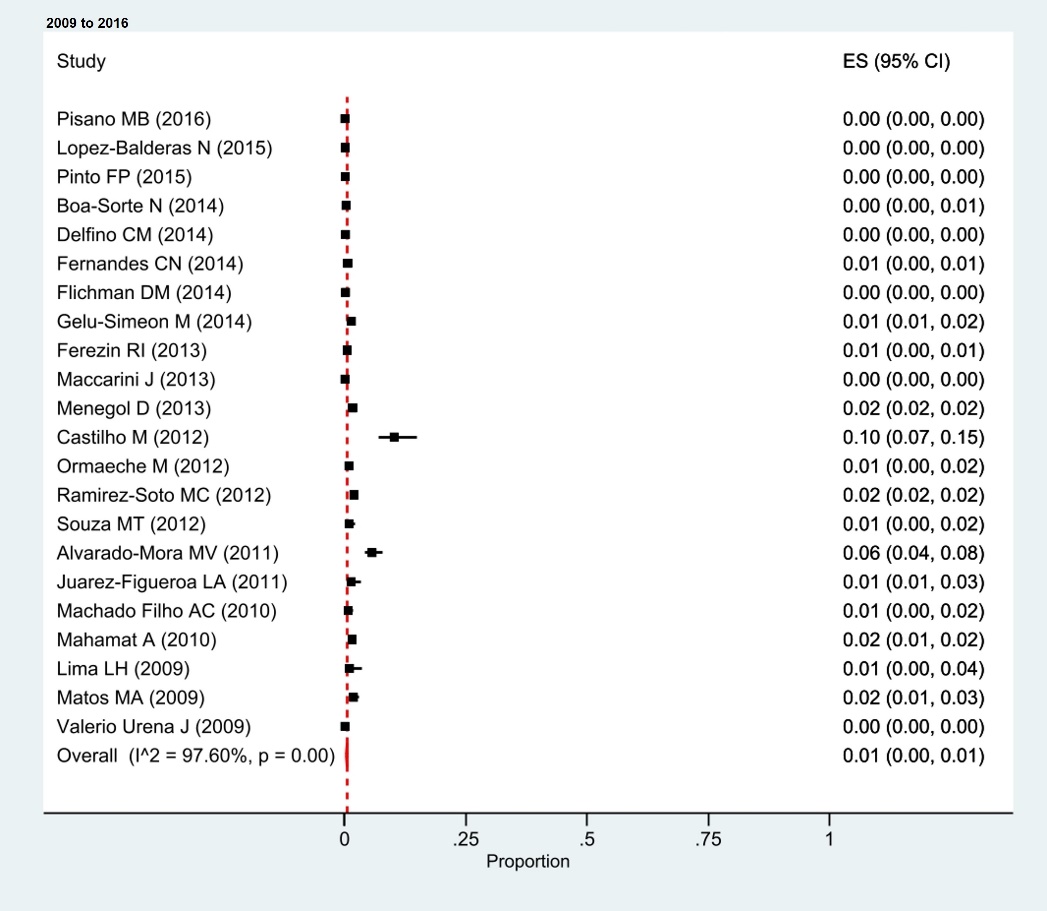


**(C)** 2017 to 2024

**
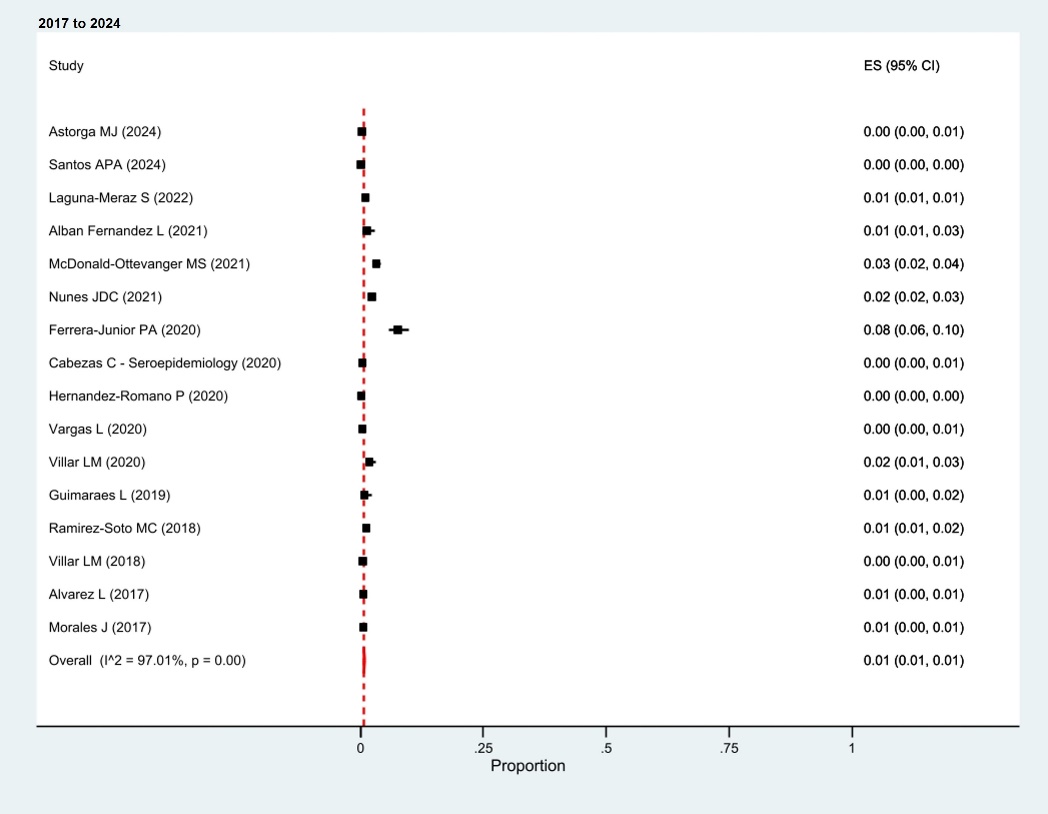
**

**Supplementary Material S9.** Forest plot showing the estimated prevalence of HBV in the indigenous people of Latin America and the Caribbean, according to publication period: **(A)** 2000 to 2008, **(B)** 2009 to 2016, and **(C)** 2017 to 2024.

**(A)** 2000 to 2008


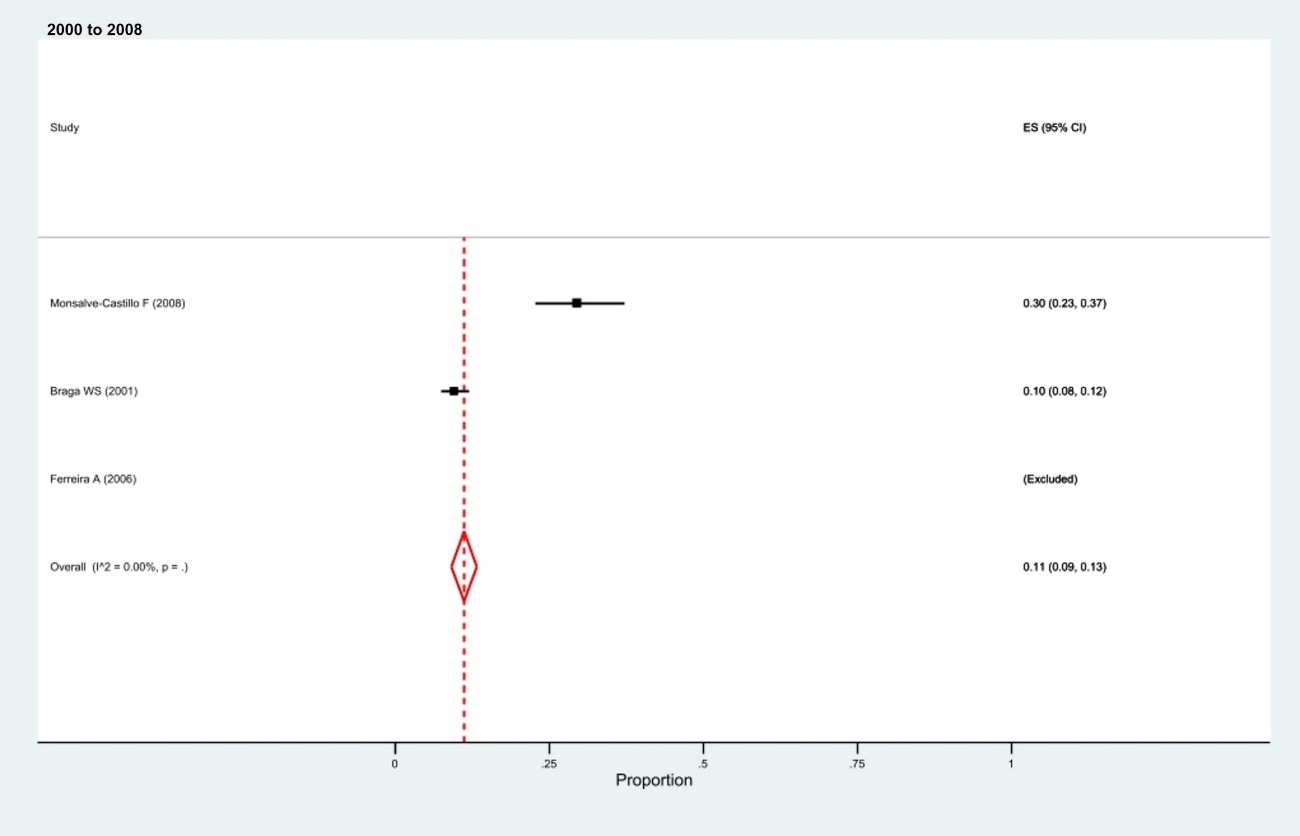


**(B)** 2009 to 2016


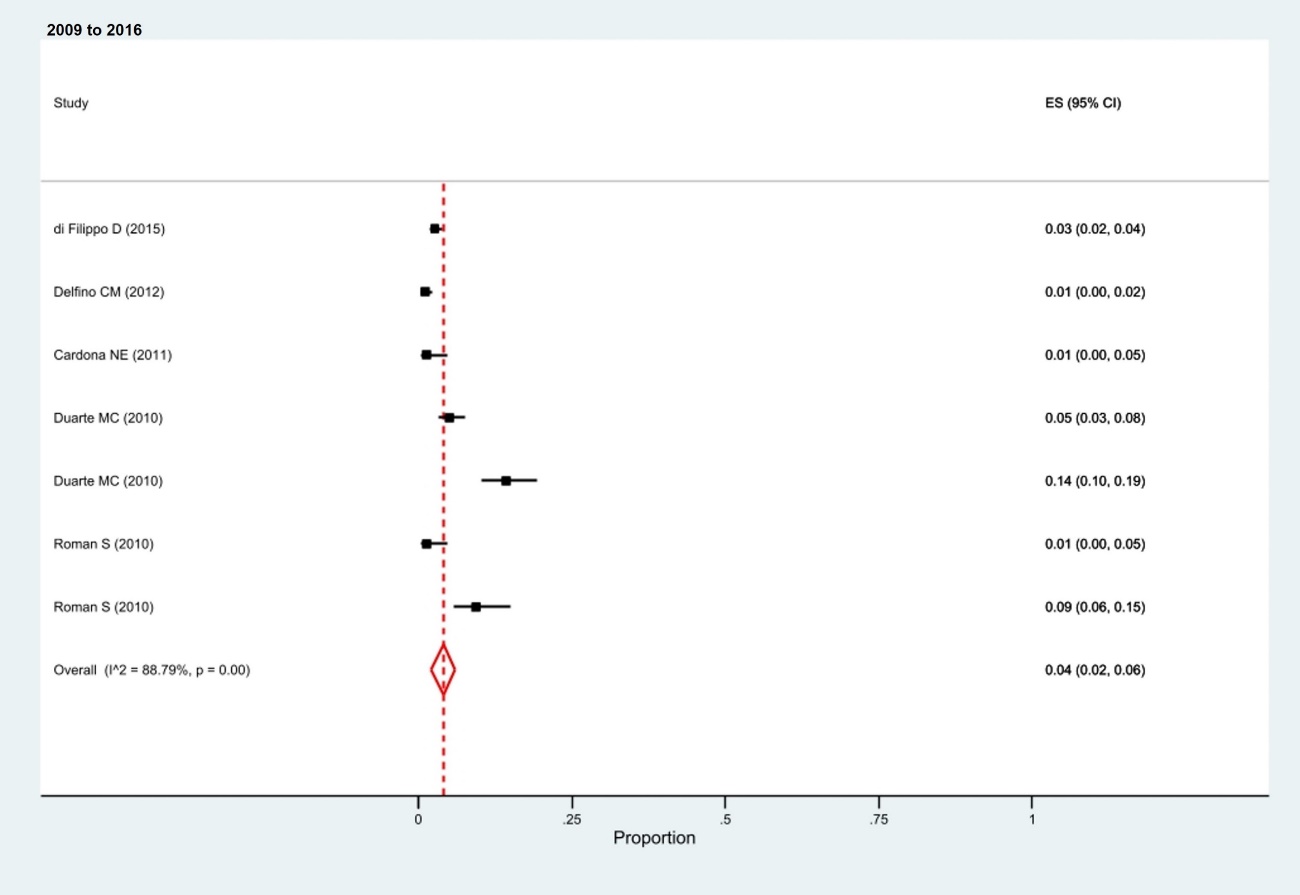


**(C)** 2017 to 2024


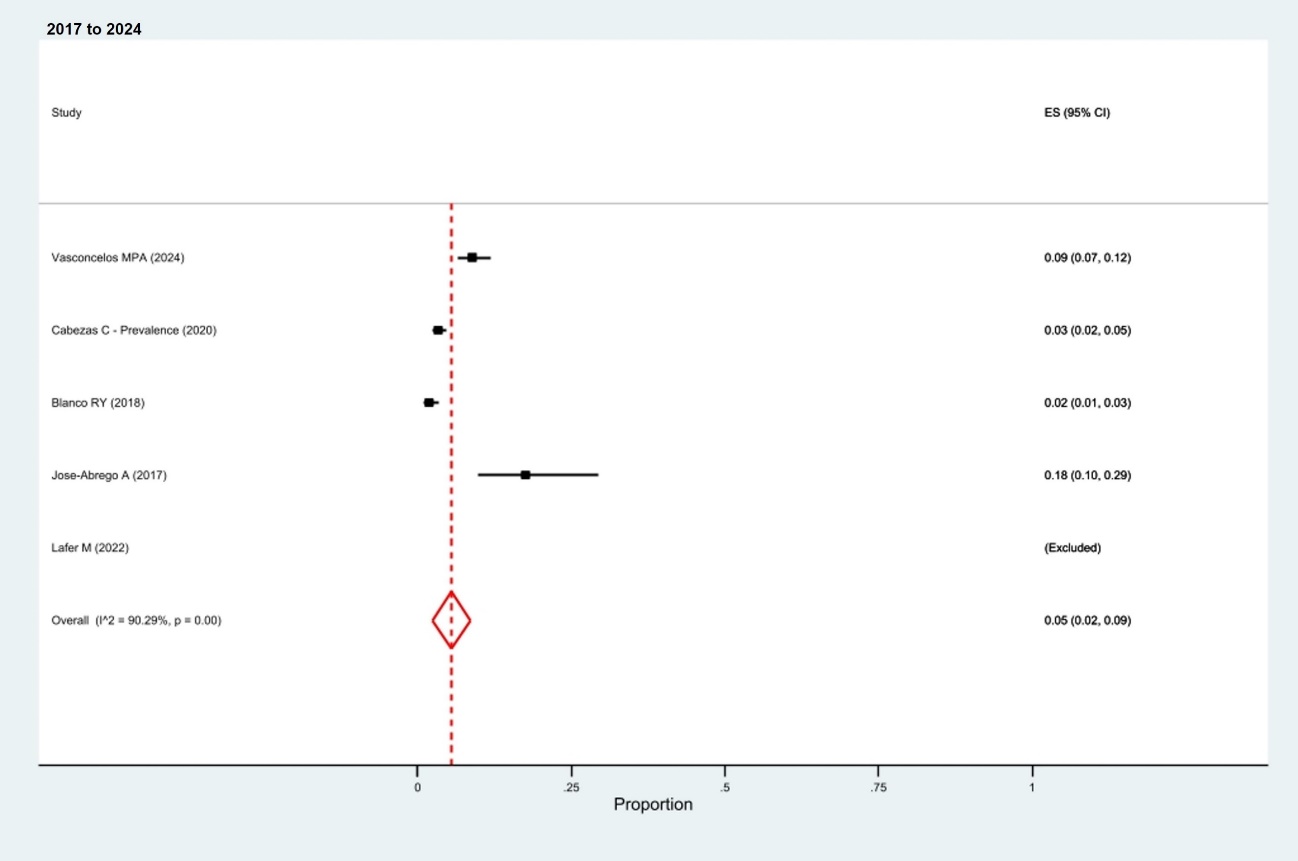


**Supplementary Material S10.** Forest plot showing the estimated prevalence of HBV in the HIV-infected individuals of Latin America and the Caribbean, according to publication period: **(A)** 2000 to 2008, **(B)** 2009 to 2016, and **(C)** 2017 to 2024.

**(A)** 2000 to 2008


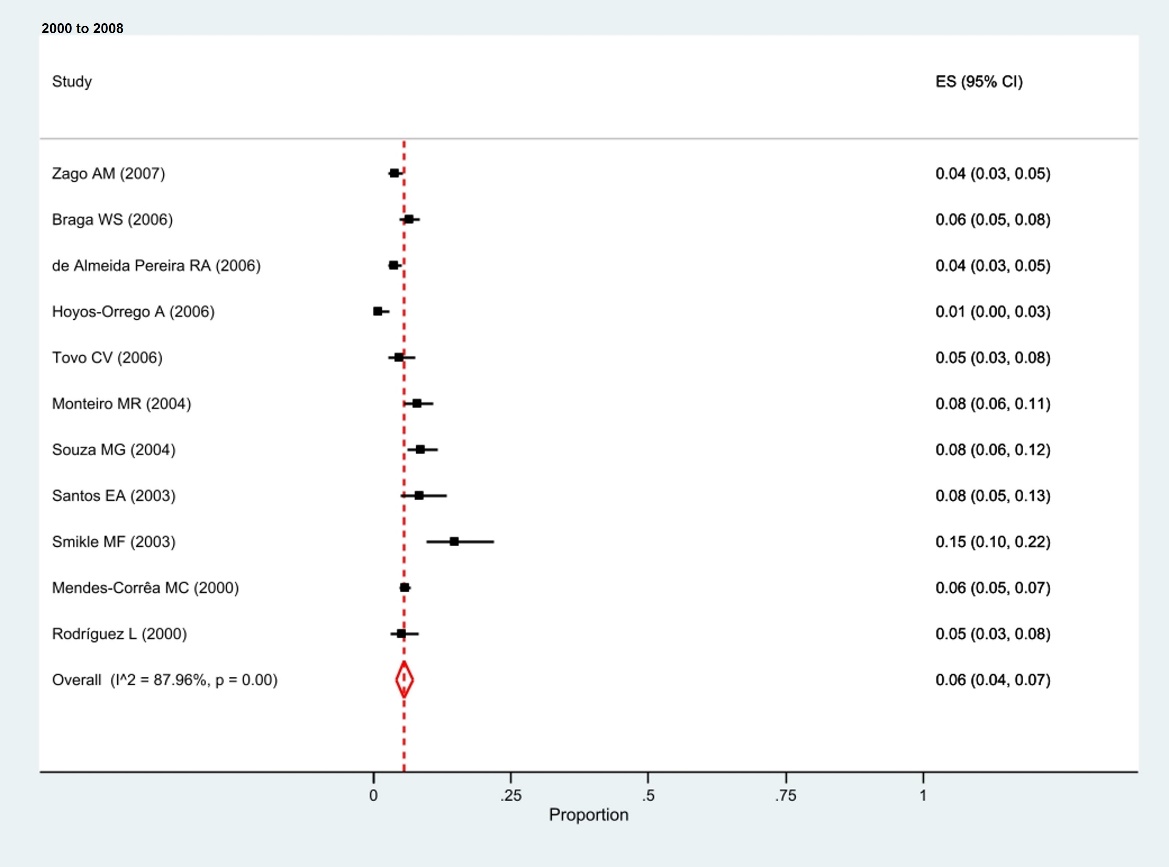


**(B)** 2009 to 2016


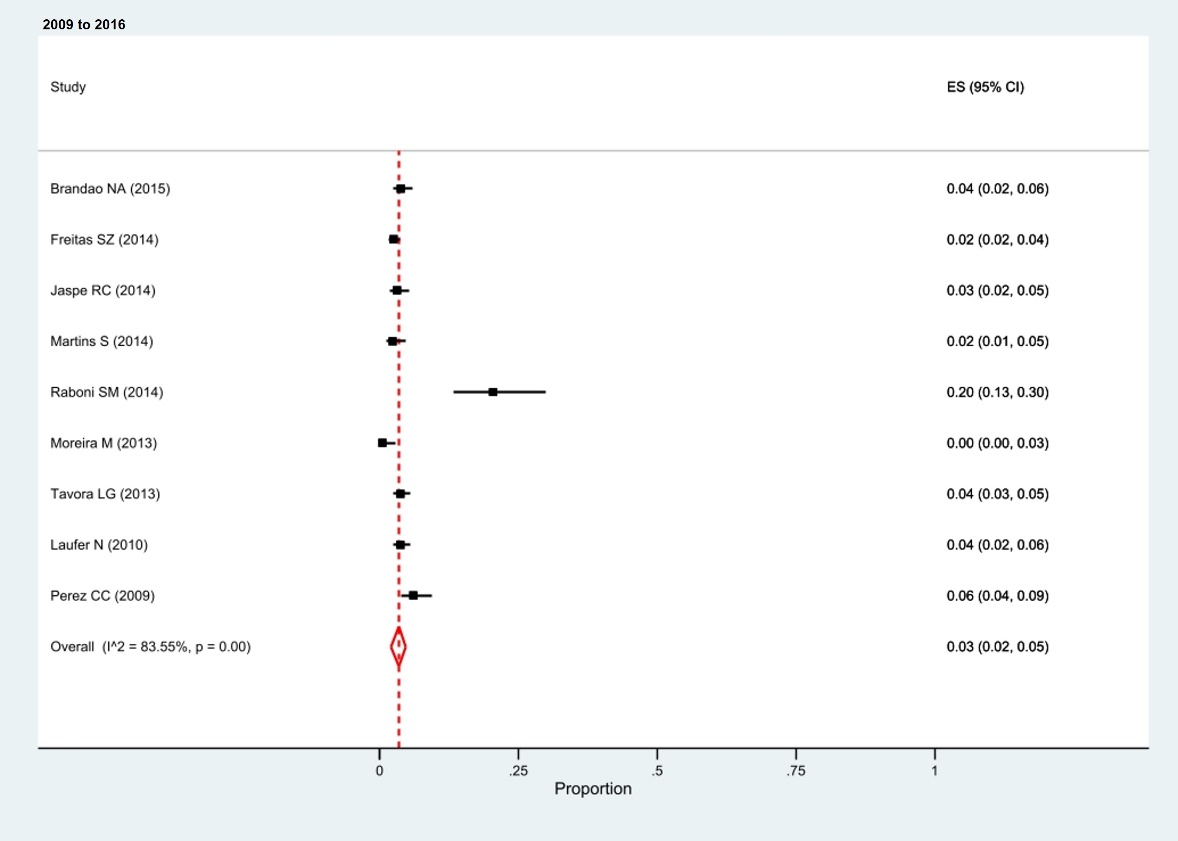


**(C)** 2017 to 2024


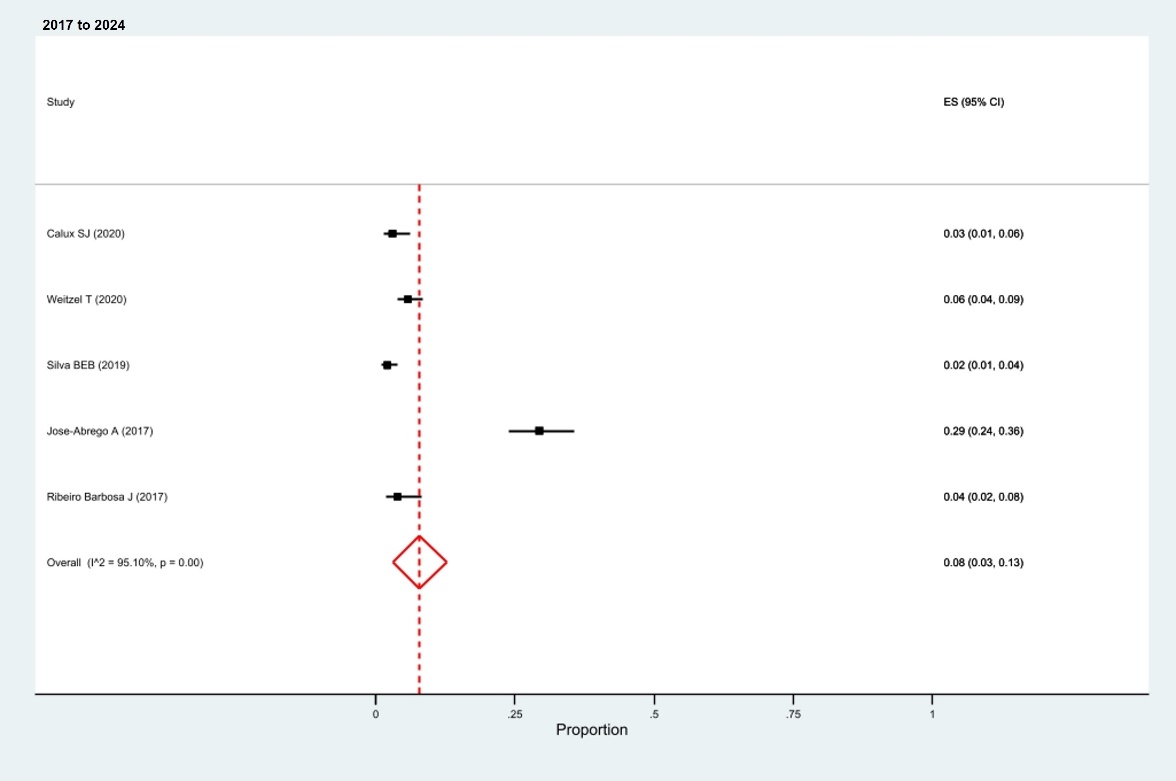


**Supplementary Material S11.** Forest plot showing the estimated prevalence of HBV in the inmates of Latin America and the Caribbean, according to publication period: **(A)** 2000 to 2008, **(B)** 2009 to 2016, and **(C)** 2017 to 2024.

**(A)** 2000 to 2008


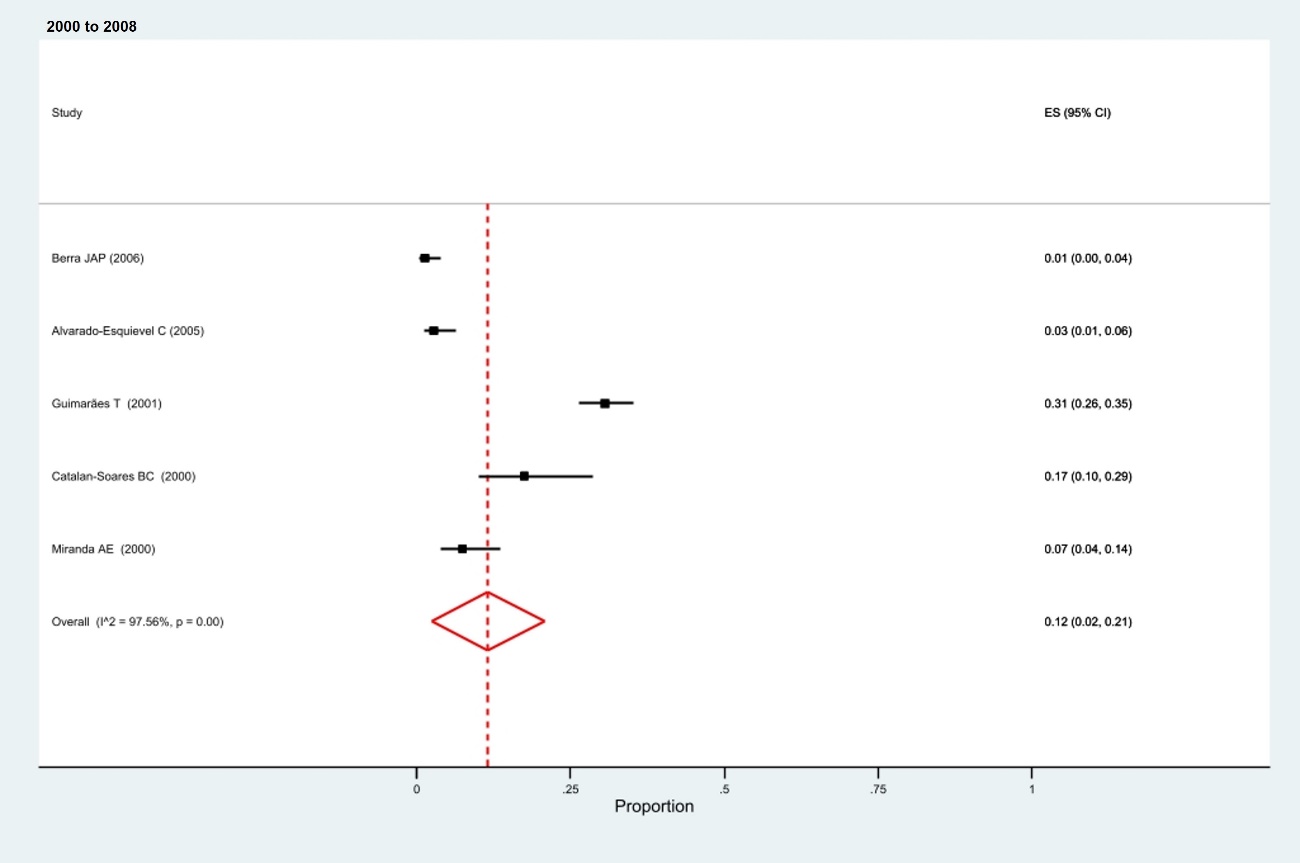


**(B)** 2009 to 2016


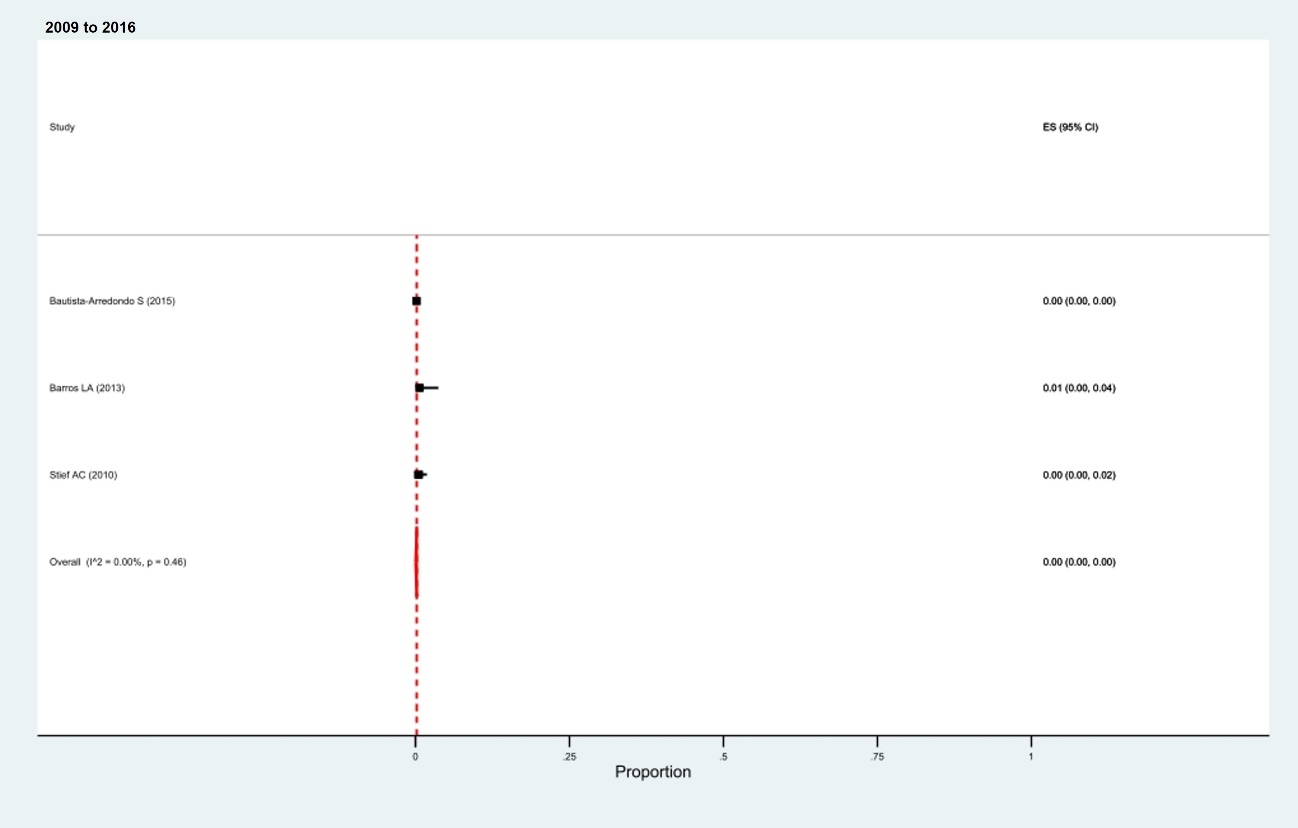


**(C)** 2017 to 2024


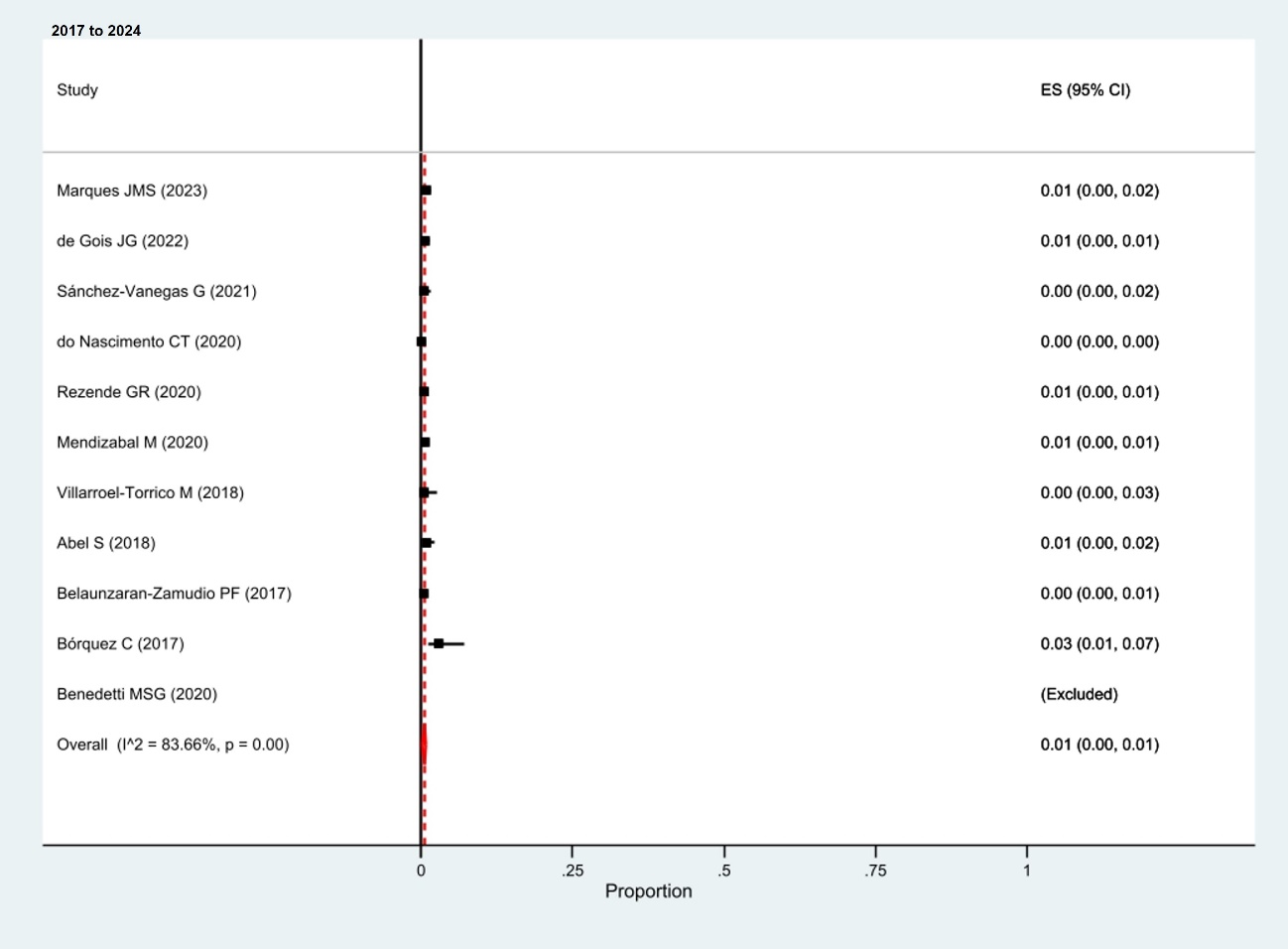


**Supplementary File S12.** Full list of references included in the meta-analysis on the prevalence of HBV in Latin America and the Caribbean.

Abel S, Cuzin L, da Cunha S, et al. Reaching the WHO target of testing persons in jails in prisons will need diverse efforts and resources. *PLoS One* 2018; 13(8): e0202985.

Aguiar JI, Aguiar E, Paniago A, Cunha R, Galvão L, Daher R. Prevalence of antibodies to hepatitis B core antigen in blood donors in the middle West region of Brazil. *Mem Inst Oswaldo Cruz* 2001; 96(2): 185-187.

Aires RS, Matos MA, Lopes CL, et al. Prevalence of hepatitis B virus infection among tuberculosis patients with or without HIV in Goiânia City, Brazil. *J Clin Virol* 2012; 54(4): 327-331.

Albán Fernández L, Albán Olaya M, López Chegne N, Rabanal Becerra D, Araujo Salazar V, Cabrera Huamán K. Seroprevalence of hepatitis B in adult population of a district of Cajamarca. *Rev Gastroenterol Peru* 2021; 41(1): 16-20.

Almeida D, Tavares-Neto J, Vitvitski L, et al. Serological markers of hepatitis A, B and C viruses in rural communities of the semiarid Brazilian northeast. *Braz J Infect Dis* 2006; 10(5): 317-321.

Almeida D, Tavares-Neto J, Trepo C, et al. Occult B infection in the Brazilian northeastern region: a preliminary report. *Braz J Infect Dis* 2008; 12(4): 310-312.

Alvarado-Esquivel C, Sablon E, Martínez-García S, Estrada-Martínez S. Hepatitis virus and HIV infections in inmates of a state correctional facility in Mexico. *Epidemiol Infect* 2005; 133(4): 679-685.

Alvarado-Mora MV, Fernandez MF, Gomes-Gouvêa MS, de Azevedo Neto RS, Carrilho FJ, Pinho JR. Hepatitis B (HBV), hepatitis C (HCV) and hepatitis delta (HDV) viruses in the Colombian population-how is the epidemiological situation? *PLoS One* 2011; 6(4): e18888.

Alvarez L, Tejada-Llacsa PJ, Melgarejo-García G, Berto G, Montes Teves P, Monge E. Hepatitis B and C prevalence in a blood bank at general hospital in Callao, Peru. *Rev Gastroenterol Peru* 2017; 37(4): 346-349.

Andrade AF, Oliveira-Silva M, Silva SG, Motta IJ, Bonvicino CR. Seroprevalence of hepatitis B and C virus markers among blood donors in Rio de Janeiro, Brazil, 1998-2005. *Mem Inst Oswaldo Cruz*. 2006; 101(6): 673-676.

Andrade AP, Pacheco SDB, Silva FQ, et al. Characterization of hepatitis B virus infection in illicit drug users in the Marajó Archipelago, northern Brazil. *Arch Virol* 2017; 162(1): 227-233.

Aquino JA, Pegado KA, Barros LP, Machado LF. Seroprevalence of hepatitis B virus and hepatitis C virus infections among individuals in the State of Pará. *Rev Soc Bras Med Trop* 2008; 41(4): 334-337.

Araujo MP, Kleine HT, Parmigiano TR, et al. Prevalence of sexually transmitted diseases in female athletes in São Paulo, Brazil. *Einstein (Sao Paulo)* 2014; 12(1): 31-35.

Assis SB, Valente JG, Fontes CJ, Gaspar AM, Souto FJ. Prevalence of hepatitis B viral markers in children 3 to 9 years old in a town in the Brazilian Amazon. *Rev Panam Salud Publica* 2004; 15(1): 26-34.

Astorga MJ, Sandoval A, Espinoza S. Seroprevalence of Hepatitis B virus in pregnancy women at the time of delivery. *Andes Pediatr* 2024; 95(2): 159-164.

Barros LA, Pessoni GC, Teles SA, et al. Epidemiology of the viral hepatitis B and C in female prisoners of Metropolitan Regional Prison Complex in the State of Goiás, Central Brazil. *Rev Soc Bras Med Trop* 2013; 46(1): 24-29.

Batista SM, Andreasi MS, Borges AM, et al. Seropositivity for hepatitis B virus, vaccination coverage, and vaccine response in dentists from Campo Grande, Mato Grosso do Sul, Brazil. *Mem Inst Oswaldo Cruz* 2006; 101(3): 263-267.

Bautista-Arredondo S, González A, Servan-Mori E, et al. A Cross-Sectional study of prisoners in Mexico City comparing prevalence of transmissible infections and chronic diseases with that in the ceneral population. *PLoS One* 2015; 10(7): e0131718.

Belaunzaran-Zamudio PF, Mosqueda-Gomez JL, Macias-Hernandez A, Rodríguez-Ramírez S, Sierra-Madero J, Beyrer C. Burden of HIV, syphilis, and hepatitis B and C among inmates in a prison state system in Mexico. *AIDS Res Hum Retroviruses* 2017; 33(6): 524-533.

Bellíssimo-Rodrigues WT, Machado AA, Bellíssimo-Rodrigues F, Nascimento MP, Figueiredo JF. Prevalence of hepatitis B and C among Brazilian dentists. *Infect Control Hosp Epidemiol* 2006; 27(8): 887-888.

Benedetti MSG, Nogami ASA, Costa BBD, et al. Sexually transmitted infections in women deprived of liberty in Roraima, Brazil. *Rev Saude Publica* 2020; 54:105.

Berra JAP, Bacetti LB, Buzo AA. Seroprevalence of HIV, syphilis, and hepatites B and C among women confined at Centro de Ressocialização Feminino of Rio Claro, São Paulo. *Rev Inst Adolfo Lutz* 2006; 65(2): 133-136.

Bertolini DA, Pinho JR, Saraceni CP, Moreira RC, Granato CF, Carrilho FJ. Prevalence of serological markers of hepatitis B virus in pregnant women from Paraná State, Brazil. *Braz J Med Biol Res* 2006; 39(8): 1083-1090.

Blanco RY, Loureiro CL, Villalba JA, et al. Decreasing prevalence of hepatitis B and absence of hepatitis C virus infection in the Warao indigenous population of Venezuela. *PLoS One* 2018; 13(5): e0197662.

Boa-Sorte N, Purificação A, Amorim T, Assunção L, Reis A, Galvão-Castro B. Dried blood spot testing for the antenatal screening of HTLV, HIV, syphilis, toxoplasmosis and hepatitis B and C: prevalence, accuracy and operational aspects. *Braz J Infect Dis* 2014; 18(6): 618-624.

Bórquez C, Lobato I, Gazmuri P, et al. Prevalence of HIV, hepatitis B virus and Treponema pallidum in inmates in the Preventive Detention Center of Arica, Chile. *Rev Chilena Infectol* 2017; 34(5): 453-457.

Bottecchia M, Miguel JC, da Silva EF, Ginuíno CF, Souza MT. Screening for hepatitis B virus in Maracanã workers. *Braz J Infect Dis* 2015; 19(1): 100-101.

Braga WS, Brasil LM, de Souza RA, Castilho M da C, da Fonseca JC. The occurrence of hepatitis B and delta virus infection within seven Amerindian ethnic groups in the Brazilian western Amazon. *Rev Soc Bras Med Trop* 2001; 34(4): 349-355.

Braga WS, da Costa Castilho M, dos Santos IC, Moura MA, Segurado AC. Low prevalence of hepatitis B virus, hepatitis D virus and hepatitis C virus among patients with human immunodeficiency virus or acquired immunodeficiency syndrome in the Brazilian Amazon basin. *Rev Soc Bras Med Trop* 2006; 39(6): 519-522.

Brandão NA, Pfrimer IA, Martelli CM, Turchi MD. Prevalence of hepatitis B and C infection and associated factors in people living with HIV in Midwestern Brazil. *Braz J Infect Dis* 2015; 19(4): 426-430.

Cabezas C, Trujillo O, Balbuena J, et al. Prevalence of retrovirus, hepatitis B and D infection in the Matsés ethnic group in Loreto, Peru. *Rev Peru Med Exp Salud Publica* 2020; 37(2): 259-264.

Cabezas C, Trujillo O, Gonzales-Vivanco Á, et al. Seroepidemiology of hepatitis A, B, C, D and E virus infections in the general population of Peru: A cross-sectional study. *PLoS One* 2020; 15(6): e0234273.

Calderón GM, González-Velázquez F, González-Bonilla CR, et al. Prevalence and risk factors of hepatitis C virus, hepatitis B virus, and human immunodeficiency virus in multiply transfused recipients in Mexico. *Transfusion* 2009; 49(10): 2200-2207.

Calux SJ, Silva VCM, Compri AP, et al. Hepatitis B: Prevalence and occult infection in HIV-infected patients. *Rev Soc Bras Med Trop* 2020; 53: e20180533.

Cardona NE, Loureiro CL, Garzaro DJ, et al. Unusual presentation of hepatitis B serological markers in an Amerindian community of Venezuela with a majority of occult cases. *Virol J* 2011; 8: 527.

Carmo RA, Melo AP, Dezanet LN, de Oliveira HN, Cournos F, Guimarães MD. Correlates of hepatitis B among patients with mental illness in Brazil. *Gen Hosp Psychiatry* 2014; 36(4): 398-405.

Carreto-Vélez MA, Carrada-Bravo T, Martínez-Magdaleno A. Seroprevalence of HBV, HCV, and HIV among blood donors in Irapuato, Mexico. *Salud Publica Mex* 2003; 45(Supp 5): S690-S693.

Castilho M da C, Oliveira CM, Gimaque JB, Leão JD, Braga WS. Epidemiology and molecular characterization of hepatitis B virus infection in isolated villages in the Western Brazilian Amazon. *Am J Trop Med Hyg* 2012; 87(4): 768-774.

Catalan-Soares BC, Almeida RT, Carneiro-Proietti AB. Prevalence of HIV-1/2, HTLV-I/II, hepatitis B virus (HBV), hepatitis C virus (HCV), Treponema pallidum and Trypanosoma cruzi among prison inmates at Manhuaçu, Minas Gerais State, Brazil. *Rev Soc Bras Med Trop* 2000; 33(1): 27-30.

Cavaretto L, Motta-Castro ARC, Teles SA, et al. Epidemiological and molecular analysis of hepatitis B virus infection in manicurists in Central Brazil. *J Med Virol* 2018; 90(2): 277-281.

Chacaltana A, Espinoza J. Seroprevalence of the infection and risk factors of hepatitis B and C in healthy military personnel. *Rev Gastroenterol Peru* 2008; 28(3): 217-225.

Ciaccia MC, Moreira RC, Lemos MF, Oba IT, Porta G. Epidemiological, serological and molecular aspects of hepatitis B and C in children and teenagers of municipal daycare facilities schools and schools in the city of Santos. *Rev Bras Epidemiol* 2014; 17(3): 588-599.

Contrera-Moreno L, de Andrade SM, Pontes ER, Stief AC, Pompilio MA, Motta-Castro AR. Hepatitis B virus infection in a population exposed to occupational hazards: firefighters of a metropolitan region in central Brazil. *Rev Soc Bras Med Trop* 2012; 45(4): 463-467.

da Silva BEB, Santos VS, Santos IER, Batista MVA, Gonçalves LLC, Lemos LMD. Prevalence of coinfections in women living with human immunodeficiency virus in Northeast Brazil. *Rev Soc Bras Med Trop* 2019; 53: e20190282.

da Silva EF, Mazo DF, Oliveira CP, Medeiros RP, Carrilho FJ, Pessôa MG. HAV and HBV seroprevalence in 1,000 patients with chronic HCV infection in a tertiary care center in São Paulo, Brazil. *Ann Hepatol* 2016; 15(5): 691-695.

de Almeida MK, Dos Santos KN, Fecury AA, et al. Prevalence of viral hepatitis B and C in riverside communities of the Tucuruí Dam, Pará, Brazil. *J Med Virol* 2012; 84(12): 1907-1912.

de Almeida Pereira RA, Mussi AD, de Azevedo e Silva VC, Souto FJ. Hepatitis B Virus infection in HIV-positive population in Brazil: results of a survey in the state of Mato Grosso and a comparative analysis with other regions of Brazil. *BMC Infect Dis* 2006; 6: 34.

de Castro Rocha DFN, da Cunha Rosa LR, de Almeida Silva C, et al. Epidemiology of HIV, syphilis, and hepatitis B and C among manual cane cutters in low-income regions of Brazil. *BMC Infect Dis* 2018; 18(1): 546.

de Gois JG, Guedes SJKO, Vieira AP, et al. Seroprevalence and factors associated with hepatitis B virus exposure in the incarcerated population from southern Brazil. *PLoS One* 2022; 17(11): e0278029.

de Paula EV, Gonçales NS, Xueref S, et al. Transfusion-transmitted infections among multi-transfused patients in Brazil. *J Clin Virol* 2005; 34(Suppl 2): S27-S32.

Delfino CM, Berini C, Eirin ME, et al. New natural variants of hepatitis B virus among Amerindians from Argentina with mainly occult infections. *J Clin Virol* 2012; 54(2): 174-179.

Delfino CM, Gentile EA, Castillo AI, et al. Hepatitis B virus and hepatitis D virus in blood donors from Argentina: circulation of HBsAg and reverse transcriptase mutants. *Arch Virol* 2014; 159(5): 1109-117.

di Filippo Villa D, Cortes-Mancera F, Payares E, et al. Hepatitis D virus and hepatitis B virus infection in Amerindian communities of the Amazonas state, Colombia. *Virol J* 2015; 12:172.

do Nascimento CT, Pena DZ, Giuffrida R, et al. Prevalence and epidemiological characteristics of inmates diagnosed with infectious diseases living in a region with a high number of prisons in São Paulo state, Brazil. *BMJ Open* 2020; 10(9): e037045.

dos Ramos Farías MS, Garcia MN, Reynaga E, et al. First report on sexually transmitted infections among trans (male to female transvestites, transsexuals, or transgender) and male sex workers in Argentina: high HIV, HPV, HBV, and syphilis prevalence. *Int J Infect Dis* 2011; 15(9): e635-40.

Douine M, Schaub R, Jardin H, et al. High prevalence of hepatitis B and syphilis in illegal gold miners in French Guiana. *Clin Microbiol Infect* 2019; 25(8): 1051-1053.

Duarte MC, Cardona N, Poblete F, et al. A comparative epidemiological study of hepatitis B and hepatitis D virus infections in Yanomami and Piaroa Amerindians of Amazonas State, Venezuela. *Trop Med Int Health* 2010; 15(8): 924-933.

El Khouri M, Duarte LS, Ribeiro RB, et al. Seroprevalence of hepatitis B virus and hepatitis C virus in Monte Negro in the Brazilian western Amazon region. *Clinics* 2005; 60(1): 29-36.

Espinoza Holguin M, Arteaga-Vizcaíno M, Porto L, et al. Hepatitis B in children with cancer. *Rev Gastroenterol Peru* 2006; 26(3): 259-264.

Ferezin RI, Bertolini DA, Demarchi IG. Prevalence of positive sorology for HIV, hepatitis B, toxoplasmosis and rubella in pregnant women from the northwestern region of the state of Paraná. *Rev Bras Ginecol Obstet* 2013; 35(2): 66-70.

Fernandes CN, Alves Mde M, de Souza ML, Machado GA, Couto G, Evangelista RA. Prevalence of seropositivity for hepatitis B and C in pregnant women. *Rev Esc Enferm USP* 2014; 48(1):91-98.

Ferreira A, Greca D, Tavares E, et al. Seroepidemiology of hepatitis B and C in Kaingang Indians in the south of Brazil. *Rev Panam Salud Publica* 2006; 20(4): 230-235.

Ferreira RC, Rodrigues FP, Teles SA, et al. Prevalence of hepatitis B virus and risk factors in Brazilian non-injecting drug users. *J Med Virol* 2009; 81(4): 602-609.

Ferreira-Junior PA, de-Oliveira EC, Martin TOG, et al. Prevalence of hepatitis B and D virus infection in a district of Mato Grosso, bordering Amazonas and Rondônia states. *Rev Soc Bras Med Trop* 2020; 53: e20190559.

Flichman DM, Blejer JL, Livellara BI, et al. Prevalence and trends of markers of hepatitis B virus, hepatitis C virus and human Immunodeficiency virus in Argentine blood donors. *BMC Infect Dis* 2014; 14: 218.

Frade PC, Raiol NC, da Costa LM, et al. Prevalence and genotyping of hepatitis B virus: a cross-sectional study conducted with female sex workers in the Marajó Archipelago, Brazil. *Int J STD AIDS* 2019; 30(9): 902-910.

Freitas SZ, Soares CC, Tanaka TS, et al. Prevalence, risk factors and genotypes of hepatitis B infection among HIV-infected patients in the State of MS, Central Brazil. *Braz J Infect Dis* 2014; 18(5): 473-480.

Gelu-Simeon M, Pillas V, Deloumeaux J, et al. Seroepidemiology of chronic hepatitis B and C in the French Caribbean Island of Guadeloupe. *BMC Res Notes* 2014; 7:55.

Guimarães LCC, Brunini S, Guimarães RA, et al. Epidemiology of hepatitis B virus infection in people living in poverty in the central-west region of Brazil. *BMC Public Health* 2019; 19(1): 443.

Guimarães MD, Campos LN, Melo AP, Carmo RA, Machado CJ, Acurcio Fde A; PESSOAS Project Network Group. Prevalence of HIV, syphilis, hepatitis B and C among adults with mental illness: a multicenter study in Brazil. *Braz J Psychiatry* 2009; 31(1): 43-47.

Guimarães T, Granato CF, Varella D, Ferraz ML, Castelo A, Kallás EG. High prevalence of hepatitis C infection in a Brazilian prison: identification of risk factors for infection. *Braz J Infect Dis* 2001; 5(3): 111-118.

Hernández-Romano P, Hernández-Romano J, Torres-Hernández RM, González-Jiménez B, López-Balderas N. Occult hepatitis B infections and anti-HBc prevalence at a resource-limited blood bank in Mexico. *Transfus Med* 2020; 30(5): 396-400.

Hoyos-Orrego A, Massaro-Ceballos M, Ospina-Ospina M, et al. Serological markers and risk factors for hepatitis B and C viruses in patients infected with human immunodeficiency virus. *Rev Inst Med Trop Sao Paulo* 2006; 48(6): 321-326.

Iglecias LM, Puga MA, Pompílio MA, et al. Epidemiological study of hepatitis B virus among prisoners with active tuberculosis in Central Brazil. *Int J Tuberc Lung Dis* 2016; 20(11): 1509-1515.

Jaspe RC, Sulbarán YF, Loureiro CL, et al. Genetic diversity of hepatitis B virus and hepatitis C virus in human immunodeficiency virus type 1-co-infected patients from Venezuela. *J Med Microbiol* 2014; 63(8): 1099-1104.

Johnston LG, Vaillant TC, Dolores Y, Vales HM. HIV, hepatitis B/C and syphilis prevalence and risk behaviors among gay, transsexuals and men who have sex with men, Dominican Republic. *Int J STD AIDS* 2013; 24(4): 313-321.

Jose-Abrego A, Panduro A, Fierro NA, Roman S. High prevalence of HBV infection, detection of subgenotypes F1b, A2, and D4, and differential risk factors among Mexican risk populations with low socioeconomic status. *J Med Virol* 2017; 89(12): 2149-2157.

Juárez-Figueroa LA, Uribe-Salas FJ, Conde-González CJ, Sánchez-Alemán MÁ. Serological markers of hepatitis B and C, and HIV in La Calera and Cuambio, Guerrero, México. *Salud Publica Mex* 2011; 53(Suppl 1): S32-S36.

Klein G, Botelho TKR, de Cordova CMM, do Livramento A. High prevalence of HBV carriers among waste collectors in the largest landfill in Latin America. *Rev Pato Trop* 2018; 47(1): 5-10.

Lafer MM, Sitnik R, Santos Júnior MSD, Rodrigues DA, Pinho JRR. Seroprevalence of hepatitis B, C and D markers in indigenous patients seen at the Native American Outpatient Clinic of Universidade Federal de São Paulo. *Einstein* *(Sao Paulo)* 2022; 20: eAO6651.

Laguna-Meraz S, Roman S, Jose-Abrego A, Sigala-Arellano R, Panduro A. A hospital-based study of the prevalence of HBV, HCV, HIV, and liver disease among a low-income population in West Mexico. *Ann Hepatol* 2022; 27(1): 100579.

Lama JR, Agurto HS, Guanira JV, et al. Hepatitis B infection and association with other sexually transmitted infections among men who have sex with men in Peru. *Am J Trop Med Hyg* 2010; 83(1): 194-200.

Laufer N, Quarleri J, Bouzas MB, Juncos G, Cabrini M, Moretti F, et al. Hepatitis B virus, hepatitis C virus and HIV coinfection among people living with HIV/AIDS in Buenos Aires, Argentina. *Sex Transm Dis* 2010; 37(5): 342-343.

Lima LH, Viana MC. Prevalence and risk factors for HIV, syphilis, hepatitis B, hepatitis C, and HTLV-I/II infection in low-income postpartum and pregnant women in Greater Metropolitan Vitória, Espírito Santo State, Brazil. *Cad Saude Publica* 2009; 25(3): 668-676.

Livramento A, Cordova CM, Spada C, Treitinger A. Seroprevalence of hepatitis B and C infection markers among children and adolescents in the southern Brazilian region. *Rev Inst Med Trop Sao Paulo* 2011; 53(1): 13-17.

Lopez-Balderas N, Bravo E, Camara M, Hernandez-Romano P. Seroprevalence of hepatitis viruses and risk factors in blood donors of Veracruz, Mexico. *J Infect Dev Ctries* 2015; 9(3): 274-282.

Maccarini Jde L, Nazario CA, Ferreira Jdos S, et al. Positive serology for viral hepatitis and donor self-exclusion in Southern Brazil. *Rev Soc Bras Med Trop* 2013; 46(4): 403-410.

MacDonald-Ottevanger MS, Boyd A, Prins M, van der Helm JJ, et al. Differences in prevalence of hepatitis B virus infection and genotypes between ethnic populations in Suriname, South America. *Virology* 2021; 564: 53-61.

Machado Filho AC, Sardinha JF, Ponte RL, Costa EP, da Silva SS, Martinez-Espinosa FE. Prevalence of infection for HIV, HTLV, HBV and of syphilis and chlamydia in pregnant women in a tertiary health unit in the western Brazilian Amazon region. *Rev Bras Ginecol Obstet* 2010; 32(4): 176-183.

Mahamat A, Louvel D, Vaz T, Demar M, Nacher M, Djossou F. High prevalence of HBsAg during pregnancy in Asian communities at Cayenne Hospital, French Guiana. *Am J Trop Med Hyg* 2010; 83(3): 711-713.

Marinho TA, Lopes CL, Teles SA, et al. Epidemiology of hepatitis B virus infection among recyclable waste collectors in central Brazil. *Rev Soc Bras Med Trop* 2014; 47(1): 18-23.

Marques JMS, Matos MA, Silva ÁMC, et al. Prevalence of overt and occult hepatitis B virus infection among an incarcerated population of Central-Western Brazil. *Acta Trop* 2023; 241: 106886.

Martins S, Livramento Ad, Andrigueti M, et al. The prevalence of hepatitis B virus infection markers and socio-demographic risk factors in HIV-infected patients in Southern Brazil. *Rev Soc Bras Med Trop* 2014; 47(5): 552-558.

Matos MA, Martins RM, da Silva França DD, et al. Epidemiology of hepatitis B virus infection in truck drivers in Brazil, South America. *Sex Transm Infect* 2008; 84(5): 386-389.

Matos MA, Reis NR, Kozlowski AG, et al. Epidemiological study of hepatitis A, B and C in the largest Afro-Brazilian isolated community. *Trans R Soc Trop Med Hy*g 2009; 103(9): 899-905.

Matos MA, França DDS, Carneiro MAS, et al. Viral hepatitis in female sex workers using the Respondent-Driven Sampling. *Rev. Saúde Pública* 2017; 51:65.

Melo LVL, Silva MA, Perdoná Gda S, et al. Epidemiological study of hepatitis B and C in a municipality with rural characteristics: Cássia dos Coqueiros, State of São Paulo, Brazil. *Rev Soc Bras Med Trop* 2015; 48(6): 674-681.

Mendes-Corrêa MC, Barone AA, Cavalheiro Nd, Tengan FM, Guastini C. Prevalence of hepatitis B and C in the sera of patients with HIV infection in São Paulo, Brazil. *Rev Inst Med Trop Sao Paulo* 2000; 42(2): 81-85.

Méndez-Sánchez N, Motola-Kuba D, Zamora-Valdés D, et al. Risk factors and prevalence of hepatitis virus B and C serum markers among nurses at a tertiary-care hospital in Mexico City, Mexico: a descriptive study. *Ann Hepatol* 2006; 5(4): 276-280.

Mendizabal M, Testa P, Rojas M, et al. Pilot study using the ECHO model to enhance linkage to care for patients with hepatitis C in the custodial setting. *J Viral Hepat* 2020; 27(12): 1430-1436.

Menegol D, Spilki FR. Seroprevalence of hepatitis B and C markers at the population level in the municipality of Caxias do Sul, southern Brazil. *Braz J Microbiol* 2014; 44(4): 1237-1240.

Miranda AE, Vargas PM, St. Louis ME, Viana MC. Sexually transmitted diseases among female prisoners in Brazil: prevalence and risk factors. *Sex Transm Dis* 2000; 27(9): 491-495.

Miranda AE, Figueiredo NC, Schmidt R, Page-Shafer K. A population-based survey of the prevalence of HIV, syphilis, hepatitis B and hepatitis C infections, and associated risk factors among young women in Vitória, Brazil. *AIDS Behav* 2008; 12(Suppl 4): S25-S31.

Miranda NTGP, de Souza RL, Monteiro JC, et al. Seroprevalence of HBV and HCV in female sex workers from four cities in the state of Pará, northern Brazil. *J Med Virol* 2021; 93(6): 3730-3737.

Monsalve-Castillo F, Echevarría JM, Atencio R, et al. High prevalence of hepatitis B infection in Amerindians in Japreira, Zulia State, Venezuela. *Cad Saude Publica* 2008; 24(5): 1183-1186.

Monteiro MR, do Nascimento MM, Passos AD, Figueiredo JF. Soroepidemiological survey of hepatitis B virus among HIV/AIDS patients in Belém, Pará-Brasil. *Rev Soc Bras Med Trop* 2004; 37(Suppl 2): 27-32.

Moraes TC, Fiaccadori FS, Souza M, et al. Hepatitis B virus infection among institutionalized mentally ill patients in Brazil. *Braz J Infect Dis* 2015; 19(6):643-647.

Morales J, Fuentes-Rivera J, Delgado-Silva C, Matta-Solís H. Viral hepatitis infection markers among blood donor in a National Hospital of Metropolitan Lima. *Rev Peru Med Exp Salud Publica* 2017; 34(3): 466-471.

Moreira M, Ramos A, Netto EM, Brites C. Characteristics of co-infections by HCV and HBV among Brazilian patients infected by HIV-1 and/or HTLV-1. *Braz J Infect Dis* 2013; 17(6):661-666.

Motta-Castro AR, Yoshida CF, Lemos ER, et al. Seroprevalence of hepatitis B virus infection among an Afro-descendant community in Brazil. *Mem Inst Oswaldo Cruz* 2003; 98(1): 13-17.

Motta-Castro AR, Martins RM, Yoshida CF, et al. Hepatitis B virus infection in isolated Afro-Brazilian communities. *J Med Virol* 2005; 77(2): 188-193.

Motta-Castro AR, Martins RM, Araujo NM, et al. Molecular epidemiology of hepatitis B virus in an isolated Afro-Brazilian community. *Arch Virol* 2008; 153(12): 2197-2205.

Motta-Castro ARC, Kerr L, Kendall C, et al. Hepatitis B prevalence among men who have sex with men in Brazil. *Trop Med Infect Dis* 2023; 8(4): 218.

Nascimento MC, Mayaud P, Sabino EC, Torres KL, Franceschi S. Prevalence of hepatitis B and C serological markers among first-time blood donors in Brazil: a multi-center serosurvey. *J Med Virol* 2008; 80(1): 53-57.

Nunes JDC, Silva DLFD, Fonseca LMB, et al. Unexpected findings of hepatitis B and delta infection in northeastern Brazil: a public health alert. *Ann Hepatol* 2021; 22: 100272.

Oliveira LH, Silva IR, Xavier BL, Cavalcanti SM. Hepatitis B infection among patients attending a sexually transmitted diseases clinic in Rio de Janeiro, Brazil. *Mem Inst Oswaldo Cruz* 2001; 96(5): 635-340.

Oliveira MP, Matos MA, Silva ÁM, et al. Prevalence, risk behaviors, and virological characteristics of hepatitis B virus infection in a group of men who have sex with men in Brazil: results from a respondent-driven sampling survey. *PLoS One* 2016; 11(8): e0160916.

Ormaeche M, Whittembury A, Pun M, Suárez-Ognio L. Hepatitis B virus, syphilis, and HIV seroprevalence in pregnant women and their male partners from six indigenous populations of the Peruvian Amazon Basin, 2007-2008. *Int J Infect Dis* 2012; 16(10): e724-30.

Pacher BM, Costa MR, Nascimento MM, Moura MC, Passos AD. Hepatitis B and C in a Brazilian deaf community. *Rev Soc Bras Med Trop* 2015; 48(5): 603-606.

Patzi-Churqui M, Terrazas-Aranda K, Liljeqvist JÅ, Lindh M, Eriksson K. Prevalence of viral sexually transmitted infections and HPV high-risk genotypes in women in rural communities in the Department of La Paz, Bolivia. *BMC Infect Dis* 2020; 20(1): 204.

Pérez CC, Cerón A I, Fuentes L G, et al. Hepatitis B, C, Treponema pallidum and Toxoplasma gondii co-infections in HIV infected patients. *Rev Med Chil* 2009; 137(5): 641-648.

Piauiense JNF, Costa CCS, Silva RJS, et al. Hepatitis B virus infection among people who use illicit drugs: prevalence, genotypes and risk factors in the State of Amapá, Northern Brazil. *Subst Use Misuse* 2020; 55(10): 1633-1639.

Pinheiro RS, Carneiro MAS, Martins RMB, et al. Hepatitis B, HIV, and syphilis in female crack cocaine users in Central Brazil. *J Assoc Nurses AIDS Care* 2017; 28(3): 438-442.

Pinto FP, Ferreira OC Jr, Olmedo DB, et al. Prevalence of hepatitis B and C markers in a population of an urban university in Rio de Janeiro, Brazil: a cross-sectional study. *Ann Hepatol* 2015; 14(6): 815-825.

Pisano MB, Blanco S, Carrizo H, Ré VE, Gallego S. Hepatitis B virus infection in blood donors in Argentina: prevalence of infection, genotype distribution and frequency of occult HBV infection. *Arch Virol* 2016; 161(10): 2813-2817.

Puga MAM, Bandeira LM, Pompilio MA, et al. Screening for HBV, HCV, HIV and syphilis infections among bacteriologically confirmed tuberculosis prisoners: an urgent action required. *PLoS One* 2019; 14(8): e0221265.

Raboni SM, Tuon FF, Beloto NC, et al. Human immunodeficiency virus and hepatitis C virus/hepatitis B virus co-infection in Southern Brazil: clinical and epidemiological evaluation. *Braz J Infect Dis* 2014; 18(6): 664-668.

Ramírez-Soto MC, Huichi-Atamari M, Aguilar-Ancori EG, Pezo-Ochoa JD. Seroprevalence of viral hepatitis B in university students in Abancay, Peru. *Rev Peru Med Exp Salud Publica* 2011; 28(3): 513-517.

Ramírez-Soto MC, Huichi-Atamari M. Hepatitis B in blood donors at a hospital in Apurimac, Peru. *Rev Peru Med Exp Salud Publica* 2012; 29(1): 163-164.

Ramírez-Soto MC, Huichi-Atamari M. Prevalence of hepatitis B and human T-lymphotropic virus infection among blood donors at a hospital in the south-central highlands of Peru. *Transfus Med* 2018; 28(3): 263-265.

Remesar M, Gamba C, Kuperman S, et al. Antibodies to hepatitis C and other viral markersin multi-transfused patients from Argentina. *J Clin Virol* 2005; 34(Suppl 2): S20-S26.

Rezende GR, Lago BV, Puga MA, et al. Prevalence, incidence and associated factors for HBV infection among male and female prisoners in Central Brazil: A multicenter study. *Int J Infect Dis* 2020; 96: 298-307.

Ribeiro Barbosa J, Sousa Bezerra C, Carvalho-Costa FA, et al. Cross-Sectional study to determine the prevalence of hepatitis B and C virus infection in high risk groups in the northeast region of Brazil. *Int J Environ Res Public Health* 2017; 14(7): 793.

Rodríguez L, Collado-Mesa F, Aragón U, Díaz B, Rivero J. Hepatitis B virus exposure in human immunodeficiency virus seropositive Cuban patients. *Mem Inst Oswaldo Cruz* 2000; 95(2): 243-245.

Rodriguez Lopes CL, Bringel Martins RM, Araújo Teles S, Silva SA, Maggi PS, Tachibana Yoshida CF. Seroepidemiological profile of hepatitis B infection in staff at hemodialysis units of Goiânia-Goiás, Central Brazil. *Rev Soc Bras Med Trop* 2001; 34(6): 543-548.

Rojas-Garcia M de J, Aguilar-Tlapale R, Montalvo-Melo MC, Sanchez-Aleman MA, Hernandez-Giron C. Seroprevalence of sexually transmitted infections in blood donors from the state blood transfusion center, Tlaxcala, Mexico. *Salud Publica Mex* 2008; 50(6): 437-438.

Roman S, Tanaka Y, Khan A, et al. Occult hepatitis B in the genotype H-infected Nahuas and Huichol native Mexican population. *J Med Virol* 2010; 82(9): 1527-1536.

Rosini N, Mousse D, Spada C, Treitinger A. Seroprevalence of HBsAg, anti-HBc and anti-HCV in Southern Brazil, 1999-2001. *Braz J Infect Dis* 2003; 7(4): 262-267.

Russi JC, Serra M, Viñoles J, et al. Sexual transmission of hepatitis B virus, hepatitis C virus, and human immunodeficiency virus type 1 infections among male transvestite comercial sex workers in Montevideo, Uruguay. *Am J Trop Med Hyg* 2003; 68(6): 716-720.

Sánchez-Vanegas G, Rodríguez-Vallejo D, Pinzón-Durán AC, Reina-Cifuentes MA, Monterrosa-Blanco A, Tiga-Segura, JA. Prevalence of syphilis, hepatitis B and human immunodeficiency virus in the male prison population in Bogotá, Colombia in 2019. *Infect* 2021; 25(2): 114-119.

Santos APA, Rios DRA, Trindade CLC, et al. Prevalence, incidence, risk factors and residual risk associated with viral infections among eligible Brazilian blood donors. *Transfus Med* 2024; 34(1): 46-53.

Santos EA, Yoshida CF, Rolla VC, et al. Frequent occult hepatitis B virus infection in patients infected with human immunodeficiency virus type 1. *Eur J Clin Microbiol Infect Dis* 2003; 22(2): 92-98.

Santos MB, Santos ADD, Silva PPD, et al. Spatial analysis of viral hepatitis and schistosomiasis coinfection in an endemic area in Northeastern Brazil. *Rev Soc Bras Med Trop* 2017; 50(3): 383-387.

Scaraveli NG, Passos AM, Voigt AR, et al. Seroprevalence of hepatitis B and hepatitis C markers in adolescents in Southern Brazil. *Cad Saude Publica* 2011; 27(4): 753-758.

Schuelter-Trevisol F, Custódio G, Silva AC, Oliveira MB, Wolfart A, Trevisol DJ. HIV, hepatitis B and C, and syphilis prevalence and coinfection among sex workers in Southern Brazil. *Rev Soc* *Bras Med Trop* 2013; 46(4): 493-497.

Silva C de O, Azevedo M da S, Soares CM, et al. Seroprevalence of hepatitis B virus infection in individuals with clinical evidence of hepatitis in Goiânia, Goiás. Detection of viral DNA and determination of subtypes. *Rev Inst Med Trop Sao Paulo* 2002; 44(6): 331-334.

Silva JLA, de Souza VSB, Vilella TAS, Domingues ALC, Coêlho MRCD. HBV and HCV serological markers in patients with the hepatosplenic form of mansonic schistosomiasis. *Arq Gastroenterol* 2011; 48(2): 124-130.

Silva PA, Fiaccadori FS, Borges AM, et al. Seroprevalence of hepatitis B virus infection and seroconvertion to anti-HBsAg in laboratory staff in Goiânia, Goiás. *Rev Soc Bras Med Trop* 2005; 38(2): 153-156.

Silva RJS, do Nascimento RS, Oliveira-Neto JAJ, et al. Detection and genetic characterization of hepatitis B and D viruses: a multi-site cross-sectional study of people who use illicit drugs in the Amazon region. *Viruses* 2021; 13(7): 1380.

Smikle MF, Dowe G, Williams EM, Thesiger C. Antibodies to hepatitis B virus and hepatitis C virus in residential detoxification clients in Jamaica. *Hum Antibodies* 2000; 9(4): 231-233.

Smikle M, Dowe G, Hylton-Kong T, Williams E. Hepatitis B and C viruses and sexually transmitted disease patients in Jamaica. *Sex Transm Infect* 2001; 77(4): 295-296.

Smikle MF, Heslop O, Vickers I, et al. A serosurvey of hepatitis B virus, hepatitis C virus, human T lymphotropic virus type-1 and syphilis in HIV-1-infected patients in Jamaica. *West Indian Med J* 2003; 52(1): 14-17.

Soares CC, Georg I, Lampe E, et al. HIV-1, HBV, HCV, HTLV, HPV-16/18, and treponema pallidum infections in a sample of Brazilian men who have sex with men. *PLoS One* 2014; 9(8): e102676.

Souto FJ, Fontes CJ, Gaspar AM. Prevalence of hepatitis B and C virus markers among malaria-exposed gold miners in Brazilian Amazon. *Mem Inst Oswaldo Cruz* 2001; 96(6): 751-755.

Souza MG, Passos AD, Machado AA, Figueiredo JF, Esmeraldino LE. HIV and hepatitis B virus co-infection: prevalence and risk factors. *Rev Soc Bras Med Trop* 2004; 37(5): 391-395.

Souza MT, Pinho TL, Santos MD, et al. Prevalence of hepatitis B among pregnant women assisted at the public maternity hospitals of São Luís, Maranhão, Brazil. *Braz J Infect Dis* 2012; 16(6): 517-520.

Stief AC, Martins RM, Andrade SM, et al. Seroprevalence of hepatitis B virus infection and associated factors among prison inmates in state of Mato Grosso do Sul, Brazil. *Rev Soc Bras Med Trop* 2010; 43(5): 512-515.

Távora LG, Hyppolito EB, Cruz JN, Portela NM, Pereira SM, Veras CM. Hepatitis B, C and HIV co-infections seroprevalence in a northeast Brazilian center. *Arq Gastroenterol* 2013; 50(4):277-280.

Toledo AC Jr, Greco DB, Felga M, Barreira D, Gadelha Mde F, Speranza FA. Seroprevalence of hepatitis B and C in Brazilian army conscripts in 2002: a cross-sectional study. *Braz J Infect Dis* 2005; 9(5): 374-383.

Tolentino YF, Fogaca HS, Zaltman C, Ximenes LL, Coelho HS. Hepatitis B virus prevalence and transmission risk factors in inflammatory bowel disease patients at Clementino Fraga Filho university hospital. *World J Gastroenterol* 2008; 14(20): 3201-3206.

Tonial GC, Passos AM, Livramento Ad, et al. Hepatitis B marker seroprevalence and vaccination coverage in adolescents in the City of Itajaí, State of Santa Catarina, Southern Brazil, in 2008. *Rev Soc Bras Med Trop* 2011; 44(4): 416-419.

Tovo CV, dos Santos DE, Mattos AZ, et al. Ambulatorial prevalence of hepatitis B and C markers in patients with human immunodeficiency virus infection in a general hospital. *Arq Gastroenterol* 2006; 43(2): 73-76.

Trenchi A, Gastaldello R, Balangero M, Irizar M, Cudolá A, Gallego S. Retrospective study of the prevalence of human T-cell lymphotropic virus-type 1/2, HIV, and HBV in pregnant women in Argentina. *J Med Virol* 2007; 79(12): 1974-1978.

Valente VB, Covas DT, Passos AD. Hepatitis B and C serologic markers in blood donors of the Ribeirão Preto Blood Center. *Rev Soc Bras Med Trop* 2005; 38(6): 488-492.

Valerio-Ureña J, Vásquez-Fernández F, Pérez-Sosa JA, et al. Prevalence of VHB and VHC serological markers among blood donors in the capital state of Veracruz, Mexico. *Gac Med Mex* 2009; 145(3): 183-187.

Vargas L, Bastos F, Guimarães A, et al. Seroprevalence and factors associated with human immunodeficiency virus, human T lymphotropic virus and hepatitis B/C infections in parturient women of Salvador - Bahia, Brazil. *Braz J Infect Dis* 2020; 24(4): 279-287.

Vasconcelos MPA, Sánchez-Arcila JC, Peres L, et al. Seroprevalence of hepatitis B, C, and D and associated factors in the semi-isolated Yanomami Amazonian indigenous community. *BMC Infect Dis* 2024; 24(1): 15.

Vázquez-Martínez JL, Coreño-Juárez MO, Montaño-Estrada LF, Attlan M, Gómez-Dantés H. Seroprevalence of hepatitis B in pregnant women in Mexico. *Salud Publica Mex* 2003; 45(3): 165-170.

Viana S, Paraná R, Moreira RC, Compri AP, Macedo V. High prevalence of hepatitis B virus and hepatitis D virus in the western Brazilian Amazon. *Am J Trop Med Hyg* 2005; 73(4): 808-814.

Villar LM, Ó KM, Scalioni LP, et al. Prevalence of hepatitis B and C virus infections among military personnel. *Braz J Infect Dis* 2015; 19(3): 285-290.

Villar LM, Milagres FAP, Lampe E, et al. Determination of hepatitis B, C and D prevalence among urban and Amerindian populations from the Eastern Brazilian Amazon: a cross sectional study. *BMC Infect Dis* 2018; 18(1): 411.

Villar LM, de Paula VS, do Lago BV, et al. Epidemiology of hepatitis B and C virus infection in Central West Argentina. *Arch Virol* 2020; 165(4): 913-922.

Villar LM, Fraga KA, Mendonça ACDF, et al. Serological and molecular characterization of hepatitis B virus infection in chronic kidney disease patients from Rio de Janeiro, Brazil. *Braz J Infect Dis* 2022; 26(3): 102371.

Villarroel-Torrico M, Montaño K, Flores-Arispe P, et al. Syphilis, human immunodeficiency virus, herpes genital and hepatitis B in a women's prison in Cochabamba, Bolivia: prevalence and risk factors. *Rev Esp Sanid Penit* 2018; 20(2): 47-54.

Vinelli E, Lorenzana I. Transfusion-transmitted infections inmulti-transfused patients in Honduras. J Clin Virol 2005; 34 (Suppl 2): S53-60.

Voigt AR, Strazer Neto M, Spada C, Treitinger A. Seroprevalence of hepatitis B and hepatitis C markers among children and adolescents in the south Brazilian region: metropolitan area of Florianópolis, Santa Catarina. *Braz J Infect Dis* 2010; 14(1): 60-65.

Weissenbacher M, Rossi D, Radulich G, et al. High seroprevalence of bloodborne viruses among street-recruited injection drug users from Buenos Aires, Argentina. *Clin Infect Dis* 2003; 37(Suppl 5): S348-S352.

Weitzel T, Rodríguez F, Noriega LM, et al. Hepatitis B and C virus infection among HIV patients within the public and private healthcare systems in Chile: A cross-sectional serosurvey. *PLoS One* 2020; 15(1): e0227776.

Zago AM, Machado TF, Cazarim FL, Miranda AE. Prevalence and risk factors for chronic hepatitis B in HIV patients attended at a sexually-transmitted disease clinic in Vitória, Brazil. *Braz J Infect Dis* 2007; 11(5): 475-478.
